# Leveraging a spectrum of cytogenomics methods for profiling complex karyotypes in chronic lymphocytic leukemia

**DOI:** 10.1186/s40246-026-00957-4

**Published:** 2026-04-11

**Authors:** Karolina Cernovska, Sabina Penazova, Kamila Stranska, Jakub Paweł Porc, Patricie Skalakova, Eva Ondrouskova, Tobias Rausch, Jan Svaton, Kristyna Tausova, Natalie Kazdova, Karol Pal, Jakub Hynst, Vladimir Benes, Marie Jarosova, Sarka Pospisilova, Jana Kotaskova, Karla Plevova

**Affiliations:** 1 Institute of Medical Genetics and Genomics, Faculty of Medicine, Masaryk University & University Hospital, Brno, Czech Republic; 2 Centre for Molecular Medicine, Central European Institute of Technology, Masaryk University, Brno, Czech Republic; 3 Department of Internal Medicine, Hematology and Oncology, University Hospital Brno & Faculty of Medicine, Masaryk University, Brno, Czech Republic; 4 Genomics Core Facility, European Molecular Biology Laboratory, Heidelberg, Germany

**Keywords:** Chronic Lymphocytic Leukemia, Complex Karyotype, Nanopore Sequencing, Optical Genome Mapping, Chromatin Conformation Capture, Short-read Sequencing, Structural Variants

## Abstract

**Background:**

A highly complex karyotype (high-CK) is a key biomarker of poor prognosis in chronic lymphocytic leukemia (CLL). While conventional methods lack the resolution to fully characterize complex structural variants (SVs), emerging technologies such as short-read WGS (sr-WGS), nanopore sequencing (ONT), optical genome mapping (OGM), and chromatin conformation capture (Micro-C) offer powerful tools for high-resolution SVs analysis, illuminating the genomic architecture underlying CLL.

**Methods:**

We selected nine CLL cases bearing diverse genomic alterations. Each underwent routine diagnostic evaluation via chromosome banding analysis (CBA), multicolor fluorescence in situ hybridization (mFISH), and chromosomal microarray (CMA) and was further analyzed using sr-WGS, ONT, OGM, and Micro-C. We performed a comprehensive comparison of copy number variants (CNVs) and SVs across all methods.

**Results:**

Across five methods (CMA, ONT, OGM, sr-WGS, Micro-C), 56.3% (179/318) of CNVs were consistently detected. A high-confidence CNV set, defined as those identified by ≥ 3 methods, comprised 70.4% (224/318). SV detection varied by genome complexity: 2910 unique breakends (BNDs) were identified, with only 10.9% (320/2910) supported by all methods. A high-confidence SV set, supported by ≥ 3 methods, included 20.4% (595/2910) of BNDs. Dicentric chromosomes (DICs) and complex derivative chromosomes (CDERs), particularly those involving BNDs near centromeric or telomeric regions, were the most difficult to resolve. Micro-C fully confirmed 71.4% (5/7) of CDERs and all ten DICs. Overall, Micro-C aligned best with classical cytogenetics, confirming 85.5% (47/55) of aberrations, followed by OGM (65.5%) and both ONT and sr-WGS (56.4%).

**Conclusion:**

Each technology offers unique insights into the leukemia genome. Combining classical cytogenetics with high-throughput methods improves the detection of structural complexity and clinically relevant alterations.

**Supplementary Information:**

The online version contains supplementary material available at 10.1186/s40246-026-00957-4.

## Introduction

In cancer cells, a broad spectrum of structural and numerical aberrations can be present. Multiple chromosomal abnormalities (typically ≥ 3) within a cell denote a complex karyotype (CK), which has been, in hematological malignancies, repeatedly associated with an adverse clinical course [1–7]. In chronic lymphocytic leukemia (CLL), recent studies evaluating responses to novel targeted therapies highlighted the prognostic significance of CK. These findings suggest that CK may serve as an independent predictor of poor clinical outcomes, regardless of *TP53* or IGHV mutational status [8–10]. The presence of five or more chromosomal aberrations, referred to as high-CK, is associated with genomic instability, therapy resistance, and inferior survival outcomes [6, 8, 11, 12]. Chromothripsis (cth), a catastrophic event leading to extensive chromosomal rearrangements, has been identified as one of the key mechanisms driving CK [9, 13]. Unlike the gradual accumulation of mutations, cth induces rapid genomic reorganization, frequently associated with tumor suppressor (e.g., *TP53, ATM*) inactivation and oncogene (e.g., *MYC, CDK4*) amplification, thereby accelerating leukemogenesis [14–16].

Methods of classical and molecular cytogenetics, such as chromosomal banding analysis (CBA) and fluorescence in situ hybridization (FISH), have been widely implemented in laboratory diagnostics [17, 18] and serve as invaluable tools for the detection of chromosomal abnormalities. However, their limitation lies in the inability to provide detailed information about the genomic sequence and restricted genome-wide coverage, particularly for complex structural variants (SVs). Chromosomal microarrays (CMA) represent another commonly used method for the identification of copy number variations (CNVs), including chromosomal gains and losses [19]. However, their limitation lies in not detecting balanced aberrations and not providing contextual insight regarding genomic rearrangements.

The integration of next-generation sequencing (NGS) into routine diagnostics has markedly enhanced the detection of critical mutations, such as those in the *TP53* gene [20]. And while NGS still faces challenges in resolving complex SVs, its increasing affordability is driving a paradigm shift from traditional cytogenetic approaches to high-resolution, sequencing-based methods [21–23]. Moreover, recent advancements in short-read whole genome sequencing (sr-WGS) have significantly enhanced the characterization of complex SVs [15, 23].

Further improvements have been warranted by implementing single-molecule techniques, including long-read sequencing [24, 25], as well as Optical Genome Mapping (OGM) [26], which leverage long DNA molecules to directly detect complex SVs, resolve repetitive regions, and span large genomic rearrangements without the need for DNA amplification. Additional insights into long-range genomic context and 3D chromatin organization can be revealed by connected-molecule strategies, such as high-throughput chromosome conformation capture methods (Hi-C) and their modifications, including Micro-C [27]. All these methods enable direct detection of complex SVs and offer a more comprehensive view of genome architecture.

In this study, we applied genome-wide cytogenomic approaches to CK-CLL cases, aiming to evaluate the performance of novel methods for more refined karyotypic analysis.

## Methods

### CLL patients, initial sample processing

The study was performed on 9 CLL cases with CK, monitored and examined at the Department of Internal Medicine, Hematology, and Oncology at the University Hospital Brno, Czech Republic. All blood samples were taken after patients provided written informed consent, in accordance with the Declaration of Helsinki, under protocols approved by the institutional ethics committee (EKV-2020–070). Samples were selected based on the cytogenetic evaluation using CBA and multicolor FISH (mFISH). An additional analysis was carried out using CMA. Standard clinico-biological parameters were collected, including molecular genetic testing for recurrent CLL gene mutations, chromosomal abnormalities, and IGHV somatic hypermutation status (Table 1). CLL B-cells were separated from peripheral blood using Ficoll-Paque PLUS (GE Healthcare, USA) gradient centrifugation coupled with RosetteSep™ Human B Cell Enrichment Cocktail (STEM Cell Technologies, Canada) and initially stored in liquid nitrogen. Flow cytometry assessed the resulting purity of CLL cells as > 95%. Separated B-cells were used for sequencing-based methods (sr-WGS, nanopore sequencing – ONT, and Micro-C), OGM, and CMA. For OGM and Micro-C, if the viability decreased < 80% after thawing, the EasySep™ Dead Cell Removal (Annexin V) Kit (STEM Cell Technologies, Canada) was applied.

**Table 1:** Clinical and molecular characteristics of the CLL patient cohort

Patient No	Gender	Age at dg [y]	Rai risk at dg	TTFT [mo]	Time to CK analysis [mo]	Lines of therapy prior CK analysis	FISH result hierarchically	*TP53* gene status	IGHV somatic hypermutation status
Pt.01	F	56	High	1	84	2	del(17p)	mut/del	U
Pt.02	M	71	Intermediate	10	18	1	del(17p)	mut/del	U
Pt.03	M	70	High	0	55	4	del(13q)	wt/del	U
Pt.04	M	72	Low	16	15	0	del(11q)	wt/int	U
Pt.05	M	72	Intermediate	18	9	0	del(17p)	del/del	U
Pt.06	F	74	Low	91	112	1	del(17p)	mut/del	M
Pt.07	M	74	Low	23	1	0	del(13q)	wt/int	M
Pt.08	M	83	High	14	3	0	del(17p)	mut/del	U
Pt.09	M	74	High	NR	1	0	normal	mut/int	U

Summary of baseline clinical information and key molecular features for the 9 patients included in this study, including gender, age, Rai stage, time to first treatment (TTFT), time to complex karyotype (CK) analysis, number of therapy lines, FISH cytogenetics, *TP53* status, and IGHV mutational status. *NR - not reached;* *mut–mutated TP53 gene; wt–wild-type TP53 gene; del–TP53 locus deleted; int–TP53 locus intact; U–unmutated IGHV; M–mutated IGHV*

### Chromosome banding analysis (CBA)

Peripheral blood samples were treated according to the stimulation protocol for metaphase induction based on CpG-oligonucleotide DSP30 plus interleukin-2 [28]. Karyotypes were captured at magnification 1000 × and documented on LUCIA Cytogenetics software (Laboratory Imaging s.r.o, Czech Republic). Karyotypes were evaluated according to the recommendations of the ISCN 2020 (International System for Human Cytogenomic Nomenclature) [29]. Patients' karyotypes with ≥ 1 clones with ≥ 3 abnormalities were evaluated as CK. A clone had to have at least two metaphases with the same aberration if the aberration was a chromosome gain or a structural rearrangement, and at least three metaphases if the abnormality was a chromosomal loss.

### Fluorescence in situ hybridization (FISH)

CBA was enhanced with the mFISH method (24XCyte, MetaSystem, Germany) for a more precise description of karyotype changes. The metaphases were captured using an Axio Imager Z2 microscope at magnification 630 × (Zeiss, Jena, Germany) and analyzed with the NEON/ISIS software (MetaSystems, Germany).

Besides, the standard CLL panel (XL *ATM*/*TP53*, XL *DLEU*/*LAMP*/12cen; MetaSystems GmbH, Germany) was used for the detection of del(11q), del(17p), del(13q), and + 12, according to the manufacturer's instructions. Signals were documented using LUCIA Cytogenetics software (Laboratory Imaging s.r.o, Czech Republic) [30]. The threshold for positive results was set at 5%.

### Chromosomal microarray analysis (CMA)

Tumor genomic DNA (gDNA) extracted using the DNeasy® Blood & Tissue Kit (Qiagen, Germany) was used for the analysis of CNVs and losses of heterozygosity (LOH) using the CMA CytoScan HD and the respective reagent bundle (Thermo Fisher Scientific, USA), as described previously [31]. Data were analyzed in the Chromosome Analysis Suite software (ChAS, version 4.5) with the NetAffx Genomic Annotation hg19 (version 20240701). Genomic abnormalities were inspected while filtering for losses > 25 kbp, gains > 50 kbp, and LOHs > 5 Mbp in a default setting and were further compared against the Database of Genomic Variants (DGV) and ChASDB aDGV healthy individuals' dataset. The final sets of presumably somatic, CLL-associated genomic abnormalities were recorded according to the ISCN 2020 nomenclature. To ensure compatibility with other methods, the data were converted to the GRCh38 reference genome using UCSC LiftOver [32].

### Short-read WGS (sr-WGS)

#### Library preparation and sequencing

The same tumor gDNA and a paired non-tumor gDNA isolated from a buccal swab (MagCore Genomic DNA Tissue Kit, RBC Bioscience, Taiwan) were analyzed for each patient. Buccal swabs were used as a source of matched healthy control DNA, following recommendations for CLL [20]. The whole-genome sequencing libraries were prepared from 25 ng (measured on Qubit® 2.0 Fluorometer; Invitrogen, USA) of fragmented gDNA using the Invitrogen™ Collibri™ PS DNA Library Prep Kit (Invitrogen, USA) for Illumina™ or NEBNext Ultra II DNA Library Prep Kit (New England Biolabs, USA) according to the manufacturer’s instructions. The libraries were sequenced on the Illumina NovaSeq 6000 platform (Illumina, USA).

#### Bioinformatic analysis

A comprehensive pipeline was developed for detecting somatic SVs and CNVs in tumor-normal paired samples from CLL patients. The workflow integrated multiple steps, from read mapping and SV calling to feature annotation and CNV analysis. The raw fastq data were mapped against the human reference genome GRCh38 using the BWA-MEM algorithm [33]. To eliminate redundant reads, PCR duplicates were identified and marked using the GATK MarkDuplicates tool (https://broadinstitute.github.io/picard/).

CNV and SV analyses were performed using the Delly variant caller (v1.3.1) [34]. For CNV analysis, the read-depth profiles were normalized, segmented (mappable windows of 10 kb), and visualized using R scripts (log₂-transformed copy number ratios). The instructions for running Delly were followed as outlined in the available documentation at https://github.com/dellytools/delly. As the Y chromosome assembly is incomplete in the GRCh38 reference, sr-WGS data were further mapped against the T2T-CHM13 human genome reference to detect CNVs on the Y chromosome. Specific regions of interest were subsequently visualized with the Figeno tool [35]. SV calling was performed in tumor-normal mode using default parameters and the option for excluding non-mappable or repetitive regions (–exclude).

### Nanopore sequencing (ONT)

#### Library preparation and sequencing

High molecular weight (HMW DNA) gDNA was isolated using chloroform-isopropanol and sheared to approximately 15–20 kb fragments to generate consistent results. Further, the samples were prepared using the Ligation Sequencing Kit SQK-LSK110 and/or SQK-LSK114 (ONT, United Kingdom). After the initial quality control on a Flongle Flow Cell, the samples were run on the PromethION Flow Cells R9.4.1, or on R10.4.1 after their release (ONT, United Kingdom). Following a 24-h sequencing period, the PromethION Flow Cell was washed and reloaded, following the Flow Cell Wash Kit (ONT, United Kingdom) protocol. Typically, two to three portions of the library were successively loaded, based on sequencing efficiency and the available library amount. For details about sample processing, see Supplementary Material.

#### Bioinformatic analysis

ONT data were analyzed through a standardized workflow comprising basecalling, mapping, variant calling, and annotation. Basecalling was conducted using Dorado software (v0.5.3 + d9af343) (https://github.com/nanoporetech/dorado), with specific models applied based on the sequencing chemistry utilized. Following mapping, CNV analysis and SV were performed using Delly (v1.2.6) against GRCh38 human genome reference [34]. The instructions for running Delly were followed as outlined in the documentation available at https://github.com/dellytools/delly. For CNV analysis of the Y chromosome, the genome was mapped against the T2T-CHM13 human genome reference, similarly to the sr-WGS data analysis. Read depth was computed in 25 kb windows, with adaptive windowing enabled and a 25 kb scan window. All remaining parameters were set to the Delly default settings. SV calling was performed in tumor-only mode (delly lr –y ont) with default parameters and the option for excluding non-mappable or repetitive regions (–exclude).

For further analysis, variants were tagged and cross-referenced against the 1000 Genomes ONT Project using Sansa (v0.2.1) [34, 36]. For BND comparison, only BNDs separated by distances > 1000 bp on the same chromosome and with sizes exceeding 1000 bp were considered. SVs were manually inspected using the Integrative Genomics Viewer (IGV) to identify potential false positives [37]. Regions of interest were further visualized using the Figeno tool [35].

### Optical genome mapping (OGM)

#### DNA isolation, labeling and mapping

Fresh frozen B cells were pelleted to 3 million cells. Ultra-high molecular weight (UHMW) gDNA was isolated from the pellets following the Bionano Prep SP-G2 Frozen Cell Pellet DNA Isolation Protocol. Labeled UHMW gDNA was loaded on a Saphyr G3.3 chip and imaged using a Saphyr instrument (Bionano Genomics, USA) to achieve ≥ 300 × effective genome coverage. The Saphyr instrument was loaded and data were collected until full chip capacity, resulting in the high coverage observed in our data.

#### Data analysis

OGM data were analyzed using the rare variant pipeline available in the Bionano Solve 3.8.1 and visualized via Bionano Access 1.8.1 software. The default filtering criteria were applied for both SV and CNV detection (Supplementary Material). All SVs and CNVs records were manually curated and filtered. Unreliable records and redundant records describing the same SV were eliminated. SVs with variant allele frequency (VAF) < 0.05 were removed from comparisons with other methods, and a minimum supporting molecule count of five was required.

### Chromatin conformation capture (Micro-C)

#### Library preparation and sequencing

Fresh frozen B cells were used to generate pellets of 2 million cells. The Micro-C library was prepared using the Dovetail® Micro-C Kit (Cantana Bio, USA); with protocol versions 1.2 or 2.0. The protocol was modified by increasing the input to two million cells and by optimizing the enzyme concentration in stage 1 for each sample based on quality control and fragmentation profiles at the end of stage 2. The library was then sequenced on an Illumina NovaSeq 6000 platform to generate > 300 million 2 × 150 bp read pairs (Illumina, USA).

#### Bioinformatic analysis

Post-sequencing, data processing followed the workflow provided by the kit manufacturer (https://micro-c.readthedocs.io/en/latest/fastq_to_bam.html). Aligned reads were then converted to Hi-C contact matrices using Juicer Tools [38], producing.hic files binned at multiple resolutions, and KR (Knight-Ruiz) normalization was applied to correct for systematic biases. For visualization in Figeno,.hic files were converted to.mcool format using Hi-C Explorer [39], with ICE (Iterative Correction and Eigenvector decomposition) normalization performed via Cooler [40].

After preparing and normalizing the contact matrices, SVs and CNVs were identified using EagleC [41] and NeoloopFinder [42] tools. All parameters were kept at their default settings. The CNV analysis was conducted with a bin size of 25 kb, while the SV analysis utilized merged data from bin sizes of 10 kb and 50 kb to enhance detection accuracy. All results provided by the tools were manually curated and verified in physical interaction maps in Juicebox visualization software [38]. SV hit was considered positive if the interaction map showed the pattern corresponding to a distinct structural rearrangement. Chromatin interaction maps were exported directly from Juicebox software, and specific regions of interest were visualized with the Figeno tool [35].

## Results

### Overview of experimental design

**Fig. 1:**
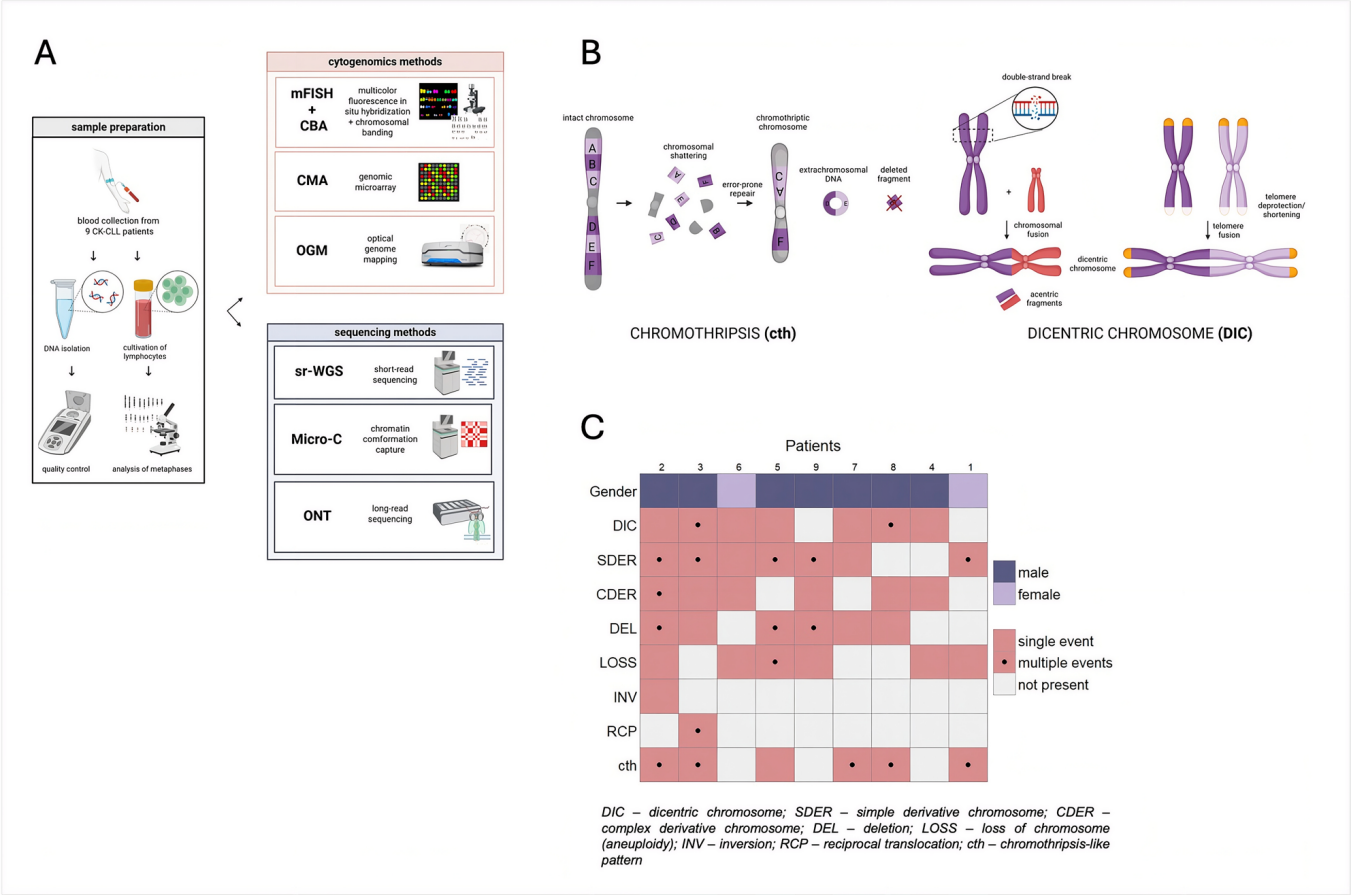
Overview of the study. **A** Study workflow. The diagram provides a visual summary of the study workflow, including sample collection, DNA isolation, and the methods evaluated in the study. The specific analytical approaches employed in this study are detailed in Supp. Figure 1A. **B** Structural aberrations observed in complex karyotype cases. *Chromothripsis* is a phenomenon in which one or more chromosomes undergo extensive chromosomal shattering due to multiple double-strand breaks, followed by error-prone repair that randomly reassembles the fragments. Unrejoined fragments may be either deleted or circularized into extrachromosomal DNA. *Dicentric chromosomes*, abnormal chromosomes containing two centromeres, formed by the fusion of chromosome segments, with or without the incorporation of acentric fragments. The diagram illustrates the possible origins of dicentric chromosomes arising from chromosome or telomere fusions. **C** Overview of the study cohort and cytogenetic assessment of CK cases. The oncoplot illustrates the distribution of gender and specific chromosomal alterations identified via mFISH; cth was reported based on CMA results. Each row corresponds to a type of SV, including simple or complex events, and cth, while each column represents an individual patient. Colored tiles indicate the presence and recurrence of events, providing an overview of the frequency and co-occurrence of events across the cohort

Routine cytogenetic analyses, including FISH and CBA, performed during the diagnostic workup identified multiple CLL cases with CK, from which nine particularly challenging cases were selected for further analyses, all of them having comprehensive data from classical cytogenomics and advanced methods, i.e., sr-WGS, ONT, Micro-C, and OGM (Fig. 1A). These cases bore chromosomal gains and losses, deletions (DELs), reciprocal translocations (RCPs), derivative chromosomes (DERs), complex chromosomal rearrangements, dicentric chromosomes (DICs), and marker chromosomes. Based on CK categorization by Baliakas et al. [8], six cases carried high-CK (≥ 5 abnormalities), whereas the remaining three low/intermediate-CK (3–4 abnormalities). Additional analysis using CMA revealed cth-like patterns in six of these cases, affecting ten different chromosomes (chr8 and chr11 were affected recurrently), and complex chromosome rearrangements (cx) on four different chromosomes (Fig. 1B). Overall, high genomic complexity (GC) as described by Leeksma et al. [43] was noted in seven cases, with the remaining two showing intermediate-GC. The full diagnostic cytogenomic characterization is summarized in Fig. 1C and Supp. Tab. 1.

Ultimately, with the genomic methods, we achieved median coverage 35.3 × for sr-WGS (range 30.1 × to 50.3 ×); 51.3 × for Micro-C (range 38.2 × to 80.8 ×); 33.7 × for ONT (range 26.8 × to 49.2 × , average N50 of 25.7 kb); and 632 × for OGM (range 298 × to 832 × , average N50 of 231.8 kb) (Supp. Figure 1B). Initially, each method was evaluated independently to establish a comprehensive list of CNVs, SVs, and corresponding breakends (BNDs) coordinates. Subsequently, we performed a comparative analysis to assess the concordance and discrepancies between the methods. Details of the data filtration strategy and method comparison criteria are provided in the Supplementary Materials.

### Comparative analysis of CNV detection across orthogonal methods.

First, we conducted a CNV comparison across all applied methods, excluding mFISH due to its intrinsic resolution limitations. When examining the number of CN changes identified by individual methods, the highest counts were reported by CMA (N = 290) and sr-WGS (N = 283), followed by ONT (N = 217), Micro-C (N = 205), and OGM (N = 192). Based on the comparison of all methods, a total of 318 BNDs were identified across the nine samples (Supp. Tab. 2, Supp. Figure 2, 4). The median number of CN changes per sample was 23, while the average reached 35. These variations reflect the differing complexity of the CK-CLL samples. Given its established use in clinical diagnostics [43, 44], CMA served as the reference method for CNV comparison. CNV calls from other methods were compared to CMA-detected CN changes and subsequently merged to unify fragmented CNVs based on predefined criteria (see Supplementary Material). To ensure consistency across all methods, we excluded any loss of heterozygosity (LOH) or absence of heterozygosity (AOH) changes from the analysis. Accurate detection of LOH/AOH events typically requires phasing information, which was not directly obtainable from all methods used in this study. Given that the primary focus of this study was on CNVs and SVs detectable by multiple independent methods comparing low- and high-throughput approaches, LOH/AOH detection was considered beyond the scope of our analysis. This approach enabled a harmonized, multi-method comparison of CNVs, while acknowledging resolution-specific constraints, ultimately improving interpretability and cross-platform comparability. Comparative summary tables (Supp. Tab. 2) provide an overview of the overlap and unique findings across all platforms for all the patients.

**Fig. 2:**
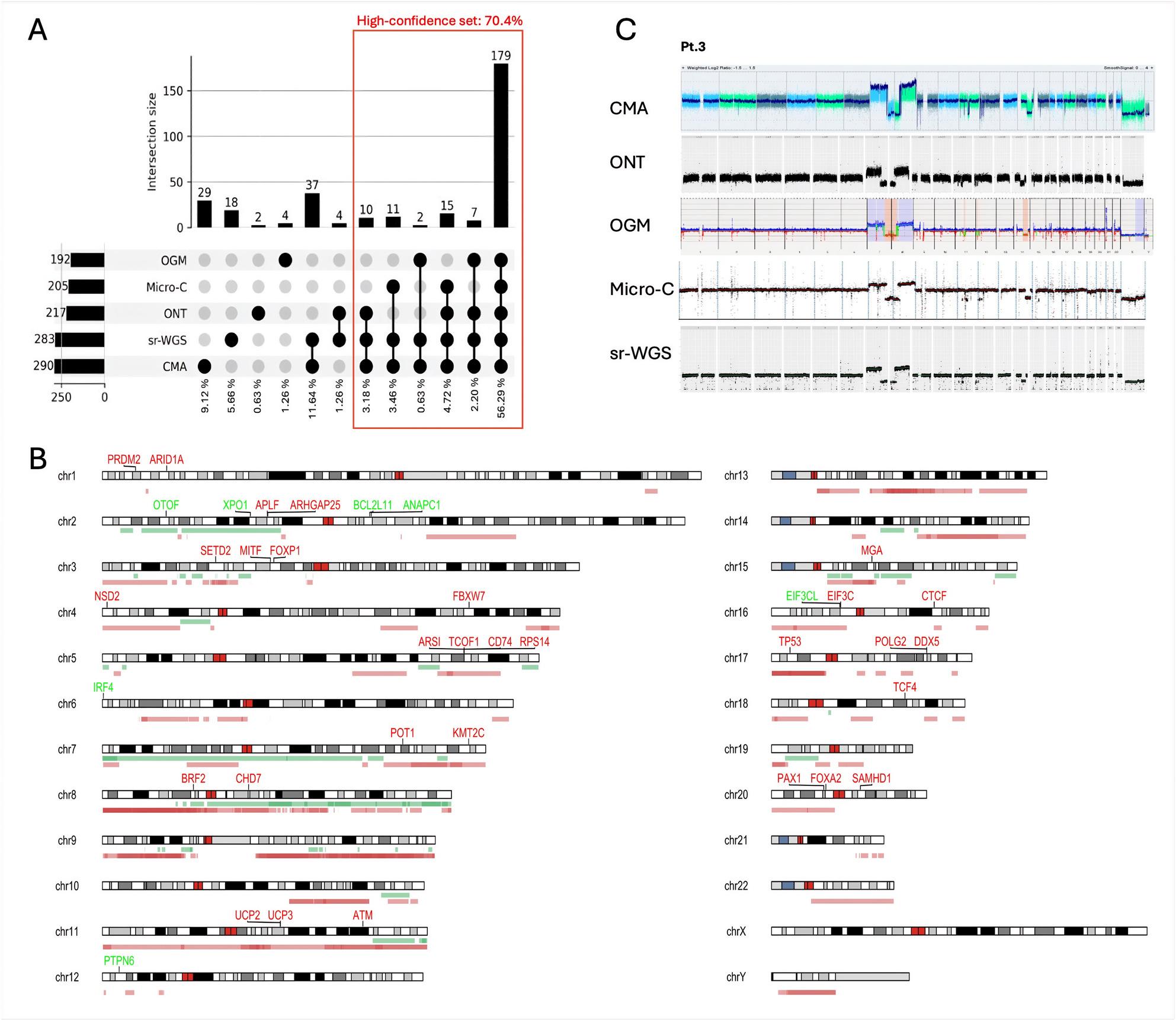
Overall results of CNV analysis from all methods across the whole cohort. **A** An Upset plot illustrating the overlap of CNV detection across different methods. Upset plots provide a quantitative representation of intersecting records, where the vertical bars indicate the number of CNVs detected by specific method combinations, while the horizontal bars represent the total CNVs detected by each method. The majority of CNVs (56,29 %) were identified by all methods, with the remaining cases distributed across various method combinations. The second most frequent category corresponds to CNVs detected by CMA and sr-WGS (11,64 %), which is likely attributable to their finer-scale resolution. **B** A schematic karyotype summarizes the CNV alterations detected across the patient cohort (N=9). Gains (in green) and losses (in red) are mapped onto chromosomal regions, with color intensity reflecting the frequency of CNV occurrences within the cohort. Additionally, genes previously reported in the literature [45, 46] as CNV-associated driver genes are annotated, with red labels indicating loss-associated and green labels indicating gain-associated genes. **C** Genome-wide CNV plots for a representative patient (Pt. 03) demonstrate a high degree of concordance among the detection methods.

Considering the size of CN change, CMA and sr-WGS uniquely detected CNVs smaller than 200 kb, while the combination of CMA, sr-WGS, and Micro-C identified CNVs in the 200–500 kb range, beyond which OGM contributed additional findings (Supp. Figure 3). To dissect the degree of concordance and method-specific biases, we generated UpSet plots representing shared and unique CNV records across the entire cohort (Fig. 2A, B). While CNV patterns exhibited considerable concordance on a broad scale (as illustrated in the example of Pt. 03 in Fig. 2C), a more detailed examination at the level of individual CNVs highlighted substantial variability in detection sensitivity and BNDs resolution across the different methods (Supp. Figure 2). Specifically, larger CNVs were detected consistently across all methods, whereas smaller CNVs showed significant discrepancies. The analysis of CNVs in the entire cohort revealed that over half of the records (179; 56.29%) were detected using all five methods. Furthermore, at least four, three, or two methods supported 201, 224, or 265 records (63.21%, 70.44%, or 83.33%), respectively. The remaining 53 reported CNVs (16.67%) were identified by either of the methods. Among the unique, method-specific variants, 47 (88.7%) were detected exclusively through CMA or sr-WGS. Overall, CMA and sr-WGS demonstrated the highest level of agreement across the methods (Fig. 2A).

Clonality of aberrations played a critical role in CNV detection. In patients with a single dominant clone (Pt. 04, 06, 07, and 09), the CNV detection overlapped across all methods, exceeding 70% concordance (Supp. Figure 2). In contrast, in cases with multiple clones (Pt. 01, 02, 03, 05, and 08), the records detected by all the methods varied between 29 and 73%, influenced by both CNV complexity and the proportional representation of the affected clones.

Furthermore, we focused on the characterization of CNVs on the sex chromosomes and loss of the Y chromosome (LOY), in particular, as it has been recognized as a risk factor in CLL [47–49]. In two patients of the cohort (Pt. 05 and 09, Supp. Figure 5), LOY was previously identified through CBA and CMA. OGM allowed the detection of LOY through the rare variant pipeline. In contrast, remapping to the T2T-CHM13 reference was required to identify LOY from sr-WGS and ONT data, as Delly with the GRCh38 reference does not support CNV analysis of chromosome Y. After remapping, both methods effectively detected the LOY. In Micro-C data, LOY could be inferred by visual inspection using Juicebox based on distinct interaction patterns (Supp. Figure 5). The incomplete Y chromosome loss in Pt. 05 was accurately resolved by CMA, sr-WGS, and ONT, which identified precise BNDs delineating the retained Y fragment. While OGM and Micro-C also indicated a LOY, they did not capture the BND and instead interpreted it as a complete loss, likely due to known challenges in analyzing the Y chromosome, such as its repetitive structure and limited mappability.

### Comparative analysis of breakend detection across orthogonal methods

In the next step, we compared the ability of the ONT, OGM, sr-WGS, and Micro-C methods to detect different SV types and assessed the sizes of the identified aberrations. We integrated SV- and CNV-derived BNDs for this purpose (Supplementary Materials). When analyzing the aberration type, we observed that sequencing-based methods, i.e., ONT, sr-WGS, and Micro-C, produced consistent aberration classification results. In contrast, OGM uses nomenclature, which recognizes more aberration categories. To facilitate a more direct comparison, we converted the OGM nomenclature to the sequencing-based classification, aligning events based on their orientation and structural characteristics.

**Fig. 3:**
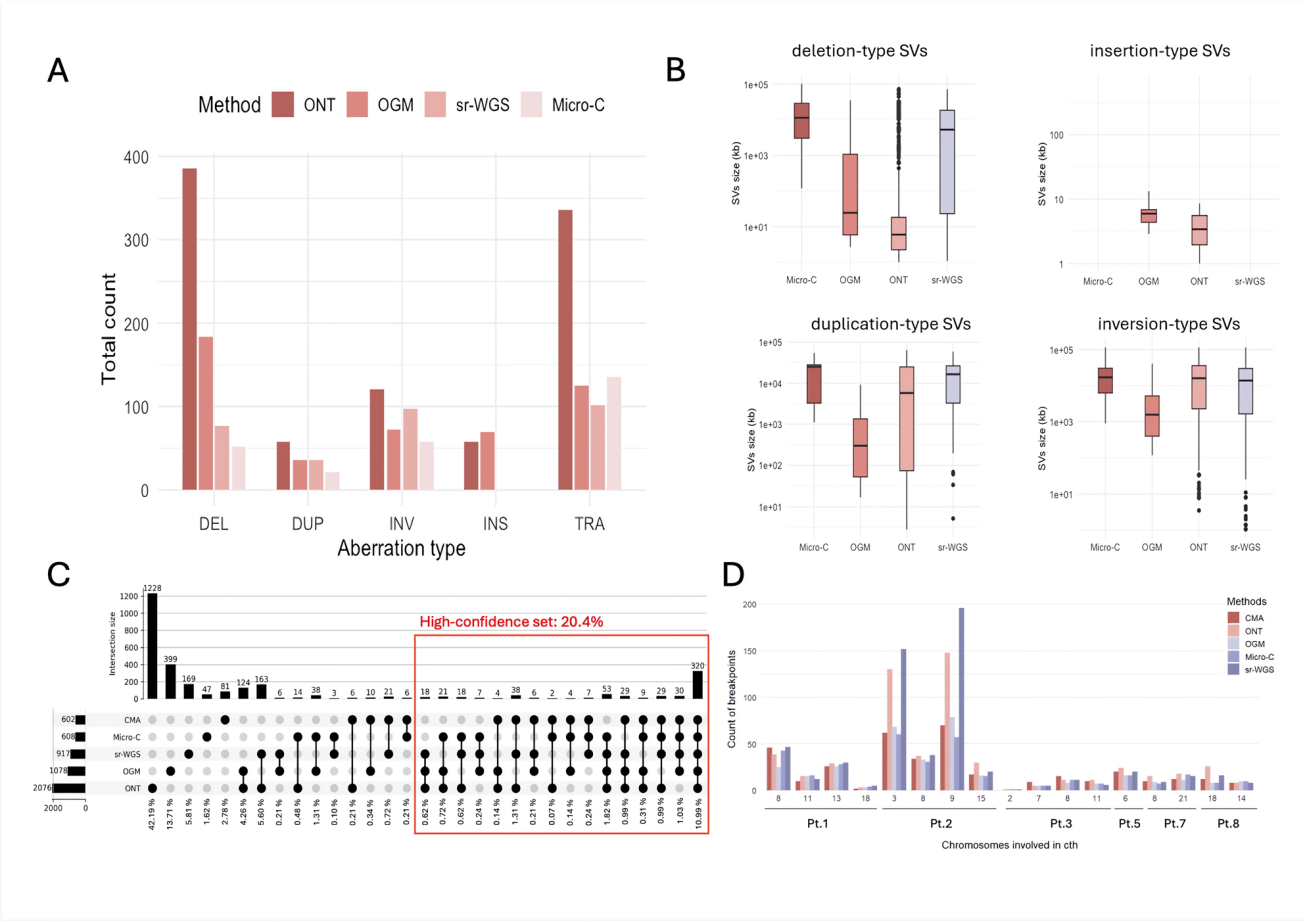
Overall results of SV analysis from all methods across the whole cohort. **A** The bar chart shows the count of each SV type detected by advanced methods (ONT, OGM, sr-WGS, and Micro-C). Insertions were exclusively detected by ONT and OGM, owing to their ability to capture long reads. *DEL–deletion-type SVs; DUP–duplication-type SVs; INV–inversion-type SVs; INS–insertion-type SVs; TRA–interchromosomal translocation-type SVs.* **B** The box plots display the sizes of each SV type detected by these methods. **C** The distribution of BNDs detected by different methods for each patient is shown in an Upset plot. Vertical bars represent the number of BNDs identified by each method, and intersecting lines indicate shared BNDs between methods. More than three methods identified 20.44% of all BNDs. **D** Chromothripsis-associated chromosomes and BND detection in designated areas by advanced methods

Notable differences were observed among the methods in the detection of various SV types and sizes, with the largest discrepancies in the number of detected events seen for DELs (Fig. 3A, B). ONT identified the highest number of DELs, with a median size of 6.1 kb. By contrast, Micro-C captured the longest deletions, with a median size of 1500 kb, whereas OGM and sr-WGS exhibited a wide range of detected DEL sizes. Inversions (INVs) were predominantly detected by ONT, while OGM and Micro-C identified similar proportions, and sr-WGS detected the fewest. Insertions (INSs) were detected exclusively by long-read capable methods, namely OGM and ONT. Notably, ONT captured INSs within a relatively small range (1–8.5 kb with median 3.4 kb), while OGM detected broader size range (2.8–610.7 kb with median 5.9 kb). Sr-WGS and Micro-C were unable to detect INSs due to the limitations of short-read sequencing. The number of detected duplications (DUPs) was relatively low compared to other SV types. (Fig. 3B).

The detection of interchromosomal translocations (TRAs) proved to be the most challenging among the SV types, especially with ONT. Despite the large number of TRAs detected by ONT, the overlap with TRAs identified by other methods was limited (Fig. 3A, Supp. Tab. 3). Manual inspection in the IGV often revealed a lack of corresponding BND on the presumed TRA partner chromosome. The issue was considerably less pronounced in sr-WGS, suggesting that the absence of a matched healthy control for ONT may have contributed to an increased number of false-positive calls. In contrast, OGM and Micro-C detected comparable counts of TRAs and demonstrated greater accuracy in TRA detection. Sr-WGS identified the fewest TRAs, although these often overlapped with those detected by OGM and Micro-C (Fig. 3A, Supp. Tab. 3).

Subsequently, we comprehensively compared BNDs identified by CMA versus ONT, OGM, sr-WGS, and Micro-C. BNDs were defined based on SV or CNV start and end positions, then merged according to predefined filtering criteria (Supplementary Material). When analyzing all BNDs identified across the entire cohort, 2910 unique BNDs were identified in total (Fig. 3C). The median number of BNDs per sample was 274 (average 323, Supp. Figure 6). ONT identified the highest number of BNDs (N = 2076), followed by OGM (N = 1078), sr-WGS (N = 917), Micro-C (N = 608), and CMA (N = 602). Importantly, 320 BNDs (10.99%) and 470 BNDs (16.15%) were supported by all applied platforms or at least four of them, respectively. Five hundred ninety-five BNDs (20.44%) were supported by at least three methods, considering these as part of our high-confidence dataset. Furthermore, 986 BNDs (33.88%) were supported by at least two methods. Conversely, 1924 BNDs (66.11%) were detected by a single method only. Of these, 1228 (62.83%) were exclusive to ONT (Fig. 3C). Given the large number of ONT-only BNDs, we investigated their genomic context using RepeatMasker. Remarkably, 95.36% of these BNDs (1171/1228) were located within or adjacent to repetitive elements. A similar proportion of repetitive BNDs (98.70%; 837/848, p = 1.351e-05) was observed among those detected by multiple methods, indicating that the repetitive nature of the region is not a distinguishing factor for BND validation (Supp. Figure 7). Among ONT-exclusive variants, smaller DELs (< 6 kb) and TRAs were the most frequent. For CMA-only and sr-WGS-only BNDs, the majority were associated with cth regions, with both methods demonstrating high sensitivity in detecting subclonal alterations often missed by other approaches. In OGM, the most unique BNDs corresponded to DELs and INSs smaller than 10 kb, with a notable proportion also associated with TRAs. Micro-C-only BNDs were predominantly TRA-associated, with the method offering robust visual validation using Juicebox for interpretation (Supp. Figure 8).

To further evaluate the performance of individual methods in complex genomic regions, we focused on cth. Six samples exhibiting cth in our cohort were identified through the presence of cth-like patterns in CMA profiles (Pt. 01 showed alterations on chromosomes 8, 11, and 13; Pt. 02 on chromosomes 3, 8, 9, and 15; Pt. 03 on chromosomes 7, 9, and 11; Pt. 05 on chromosome 6; Pt. 07 on chromosomes 8 and 21; and Pt. 08 on chromosome 18) (Supp. Tab. 5). Although ONT, OGM, sr-WGS, and Micro-C were not fully comparable across all genomic regions or in samples lacking cth chromosomes, their success rates and concordance with CMA improved markedly within cth regions. The successful confirmation rate of CMA-detected BNDs by at least one advanced method (ONT, OGM, sr-WGS or Micro-C) was 87.2% (307/352) for all cth chromosomes in the cohort (Supp. Tab. 5, Supp. Figure 9). Upon evaluating each method individually, we observed variation in the number of BNDs detected (Fig. 3D). In more complex and extensively shattered regions, such as those observed in Pt. 02 (Fig. 7), the BND counts differed. Notably, in these complex regions, all methods identified a consensus of 14.7% (83/565) BNDs, while ONT in combination with sr-WGS detected an additional 106/565 (18.8%) BNDs not identified by the other methods (Supp. Figure 9). Micro-C, on the other hand, was constrained by its predefined bin size and coverage, which limited its ability to resolve smaller aberrant regions and individual BNDs, leading to the identification of broader genomic rearrangements. For a more comprehensive analysis of cth, it is essential to incorporate directional information and precise classification of SVs. In this context, ONT, sr-WGS, and Micro-C demonstrated the highest concordance.

### Comprehensive interpretation of karyotype findings in light of genomic methods

Finally, we assessed the ability of sequencing-based approaches to detect and refine cytogenetic findings, via explicitly evaluating the location and features of abnormalities identified through CBA and mFISH. In the context of structural changes, we specifically focused on the phenomena, such as DIC and cth.

To streamline the comparison, we selected only aberrations present in at least 20% of metaphases to minimize potential differences resulting from varying coverage across the methods (Supp. Tab. 4). Across the cohort, CBA/mFISH identified 55 aberrations occurring in more than 20% cells, classified into the following classes: DELs (N = 10), reciprocal TRAs (RCPs; N = 2), chromosome losses (LOSS; N = 7), INVs (N = 1), DICs (N = 10), simple derivative chromosomes, involving 2 chromosomes and 2 BNDs (SDERs; N = 18), and complex derivative chromosomes, involving ≥ 2 chromosomes and ≥ 3 BNDs (CDERs; N = 7) (Supp. Figure 10).

#### Reevaluation of seemingly simple SVs

**Fig. 4:**
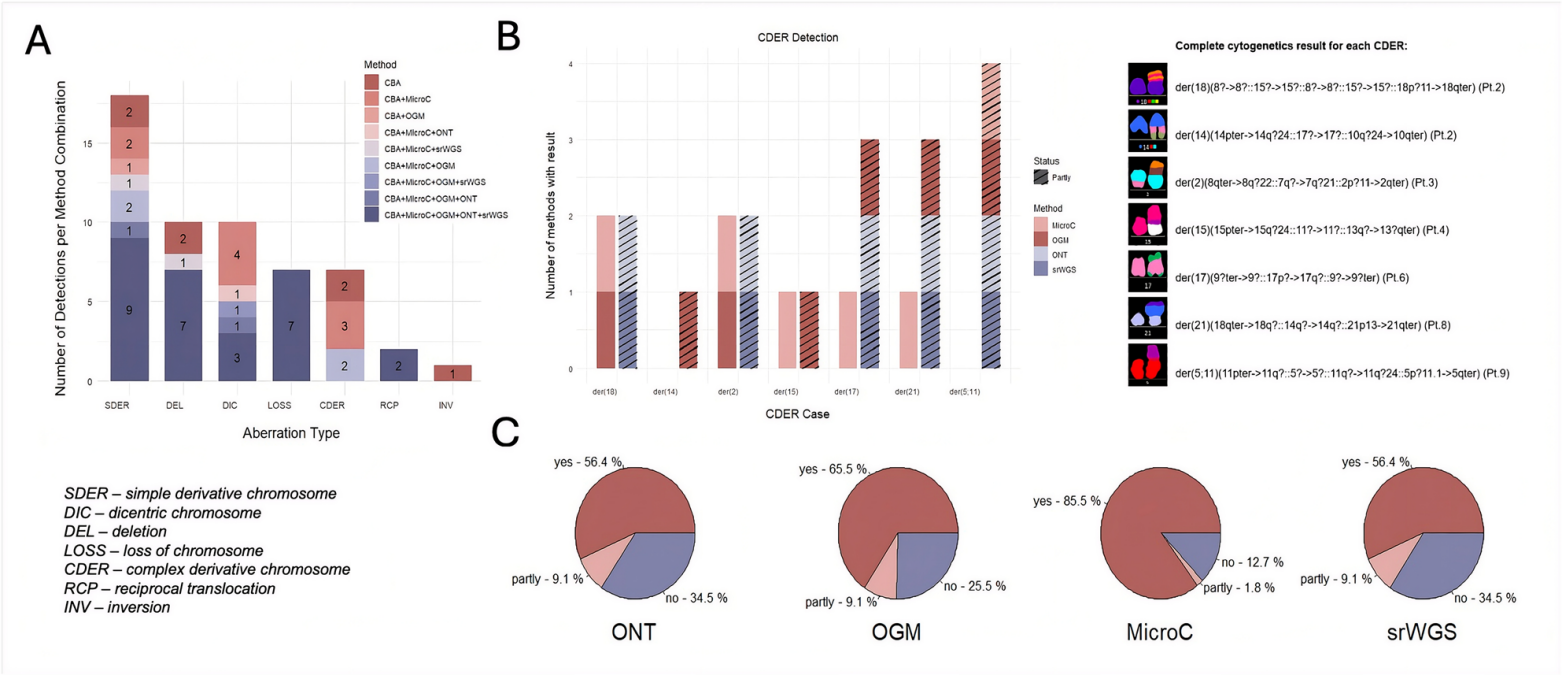
Detection rate of CBA findings using different methods. **A** Counts of aberrations initially detected by CBA in ≥ 20% of analyzed mitoses and subsequently confirmed by the tested methods and their combinations. In total, 69% of CBA-detected aberrations were validated by at least two other methods. Aberrations were classified as “yes” if confirmed, and “no” if no BND was found in the corresponding CBA-identified region. **B** However, it was not possible to validate CDER aberrations due to inconsistent findings by different methods, thus, assigned as detected “partly”. A detailed breakdown of cases classified as “partly” is shown. **C** Pie charts represent the proportion of all aberrations detected by CBA (55 aberrations in 9 patients) classified as "yes," "no," or "partly" for each advanced method

RCP and LOSS were consistently detected by all methods (Fig. 4A). The only pericentric INV present in the dataset (Pt. 03, *inv(3)(p?21;q?21)*) was not confirmed, as it was located in a highly complex genomic region (Supp. Figure 12A). ONT, OGM, sr-WGS and Micro-C instead revealed multiple INVs localized to the p-arm of chromosome 3, suggesting a substantially more complex rearrangement, consistent with a cth event on chromosome 3, rather than a simple INV. Consequently, this CBA record was classified as not confirmed. A similar pattern was observed for several DELs. While ONT, OGM, sr-WGS and Micro-C supported the majority of DELs annotated by CBA, certain cases remained unconfirmed. In these instances, the regions cytogenetically interpreted as simple DELs were revealed to harbor structurally complex rearrangements upon high-resolution analysis. This discrepancy reflects the inherent limitations of classical cytogenetics, particularly in its ability to resolve fragmented or complex chromosomal events accurately.

#### Resolving complex SVs

**Fig. 5:**
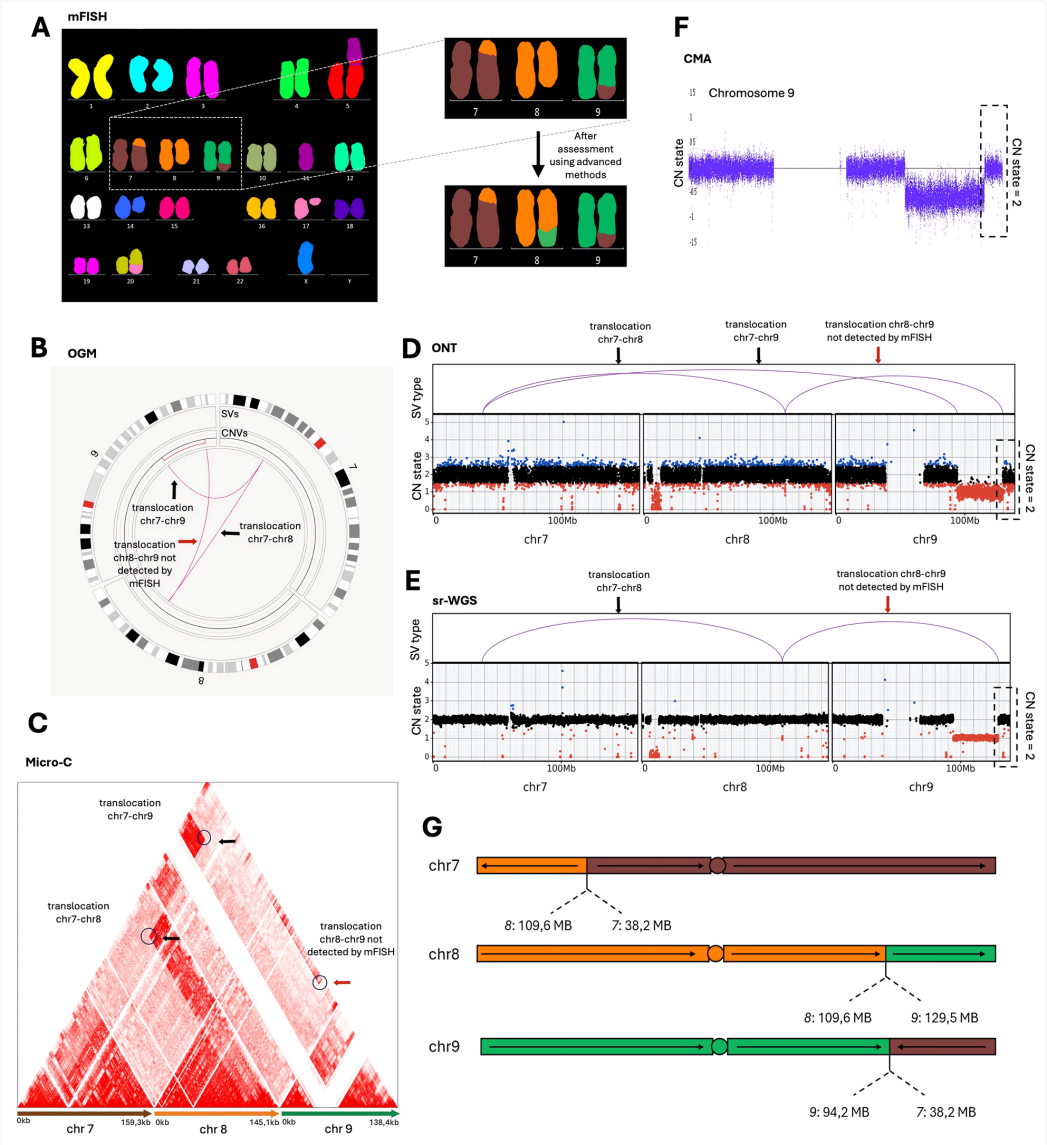
Demonstration of improved detection of rearrangements by advanced methods in Pt. 09. **A** mFISH initially identified a simple derivative chromosome 7 *(der(7)t(7;8)(p?13;q?24))* and simple derivative chromosome 9 *(der(9)t(7;9)(p?13;q?33.1))*. **B-E** All advanced genomic methods confirmed the presence of derivative chromosome 7 (black arrow). Additionally, all advanced methods, except sr-WGS, detected the derivative chromosome 9 (black arrow). **F** However, advanced methods coupled with CMA analysis revealed a normal copy number state at the terminal region of chromosome 9 (black dotted box), indicating that the missing chromosome segment was translocated elsewhere. Further analysis identified that the missing part of chromosome 9 had fused with the distal end of chromosome 8 (red arrow). **G** A schematic depiction of the structural rearranged chromosomes 7, 8, and 9.

Cases involving DIC, SDER, and CDER fell into two categories: a) when BNDs were located within interstitial regions (i.e., chromosomal arms), they were generally detected and confirmed by high-throughput methods; whereas b) when BNDs were situated in repetitive or poorly mappable regions (e.g., centromeres, telomeres, satellite DNA, and NORs – nucleolus organizer regions), the detection was often unsuccessful, limiting the ability of high-throughput methods to confirm the rearrangement. For example, the SDER *der(7)t(7;8)(p?13;q?24)* in Pt. 09. was confirmed by all methods, as both BNDs were located within mappable chromosomal arms (Fig. 5). In contrast, the SDER *der(14)t(6;14)(q?;p?11.2)* in Pt. 05 was confirmed only by Micro-C, as BNDs located in centromeric and telomeric regions could be inferred from interaction patterns observed in the interaction map (Supp. Figure 12B).

**Fig. 6:**
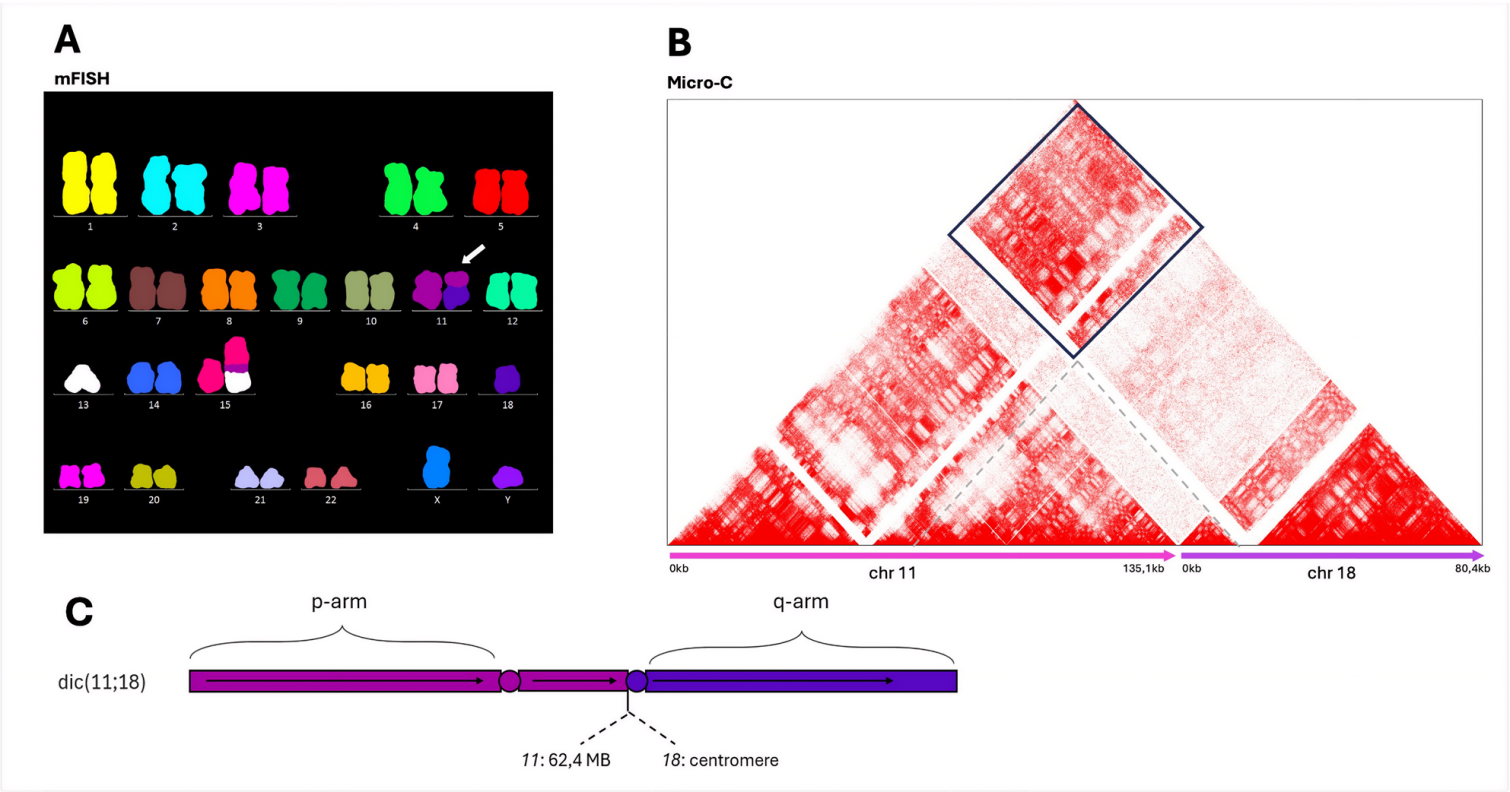
Demonstration of a dicentric chromosome identification by Micro-C in Pt. 04. **A** mFISH analysis revealed a dicentric chromosome involving chromosomes 11 and 18, designated as *dic(11;18)(?q10;?p10)*. **B** The Micro-C interaction heatmap shows increased contacts between the p-arm of chromosome 11 and the q-arm of chromosome 18, supporting the presence of the dic(11;18) rearrangement. Although the exact BNDs remain unresolved due to their proximity to centromeric regions, the altered interaction patterns provide indirect structural evidence of this rearrangement. **C** A schematic depiction of the structural rearrangement: DIC (11;18).

DIC chromosomes were among the most challenging abnormalities to detect, with the majority of the methods failing to capture them due to BNDs’ localization in centromeric or otherwise poorly mappable regions. In these cases, Micro-C proved particularly informative, offering indirect evidence of structural rearrangements through altered chromatin interaction patterns between chromosome arms, even when precise BNDs could not be resolved. For instance, in Pt. 04, CBA/mFISH identified a DIC resulting from the fusion of chromosomes 11 and 18 – *dic(11;18)(?q11;?p11)* (Fig. 6). While sr-WGS, ONT, and OGM did not detect this rearrangement, Micro-C revealed increased chromatin interactions between affected chromosomes. However, the exact BNDs on chromosome 18 could not be pinpointed, likely due to its centromeric location. This limitation was partially alleviated by ONT aligned to the T2T-CHM13 reference genome, which identified both BNDs, including the centromere-proximal site on chromosome 18. Nevertheless, these BNDs were annotated with low confidence (LowQual) in the resulting.vcf file, underscoring the persistent challenges in resolving centromeric rearrangements even with high-resolution long-read sequencing alone.

The challenge similarly applies to cases of CDERs, which consist of chromosomes with multiple BNDs, frequently involving sequences originating from two or more chromosomes (Fig. 4B). In our cohort, we identified seven cases of CDERs, highlighting their relative prevalence among patients with CK-CLL. Although sequencing-based methods and OGM have captured numerous features of these rearrangements, most CDERs had to be classified as “partly” resolved, indicating that the specific method typically identified rearrangements involving only two chromosomes, while TRAs involving the third chromosome were often missed (Fig. 4B, Supp. Figure 11). This limitation is primarily due to the localization of BNDs within repetitive genomic regions, where the current technologies encounter inherent challenges. For illustration, *der(18)(8?-* > *8?::15?-* > *15?::8?-* > *8?::15?-* > *15?::18p?11-* > *18qter)* was reported in Pt. 02, describing rearrangements between chromosomes 8 and 15 that are translocated onto chromosome 18 (Supp. Figure 12C). In this case, OGM and Micro-C successfully detected all BND events (including various TRAs between chr8 and chr15; as well as TRA between chr15 and chr18); however, sr-WGS and ONT failed to detect the aberration between chromosomes 15 and 18, given that the BND on chromosome 18 is located in the centromeric region. BND on chromosome 18 could be resolved thanks to remapping the ONT data to the T2T-CHM13 reference genome. Consistent with observations in DIC cases, Micro-C achieved the highest confirmation rate for CDERs, with 71.4% (5 cases) fully confirmed, while one case was confirmed partially, and one case remained unconfirmed. OGM followed, with 28.6% (2 cases) fully confirmed and 71.4% (5 cases) partially confirmed. In contrast, ONT and sr-WGS were unable to confirm any CDERs fully, however, each partially detected 71.4% (5 cases) (Fig. 4B, Supp.Fig. 11).

Among the evaluated methods, Micro-C demonstrated the highest overall concordance with CBA/mFISH, fully confirming 47 out of 55 aberrations (85.5%). This was followed by OGM, which fully confirmed 36 aberrations (65.5%), and both ONT and sr-WGS, each confirming 31 aberrations (56.4%) (Fig. 4A, C).

#### Demonstrating the potential of tested methods on selected cases

**Fig. 7:**
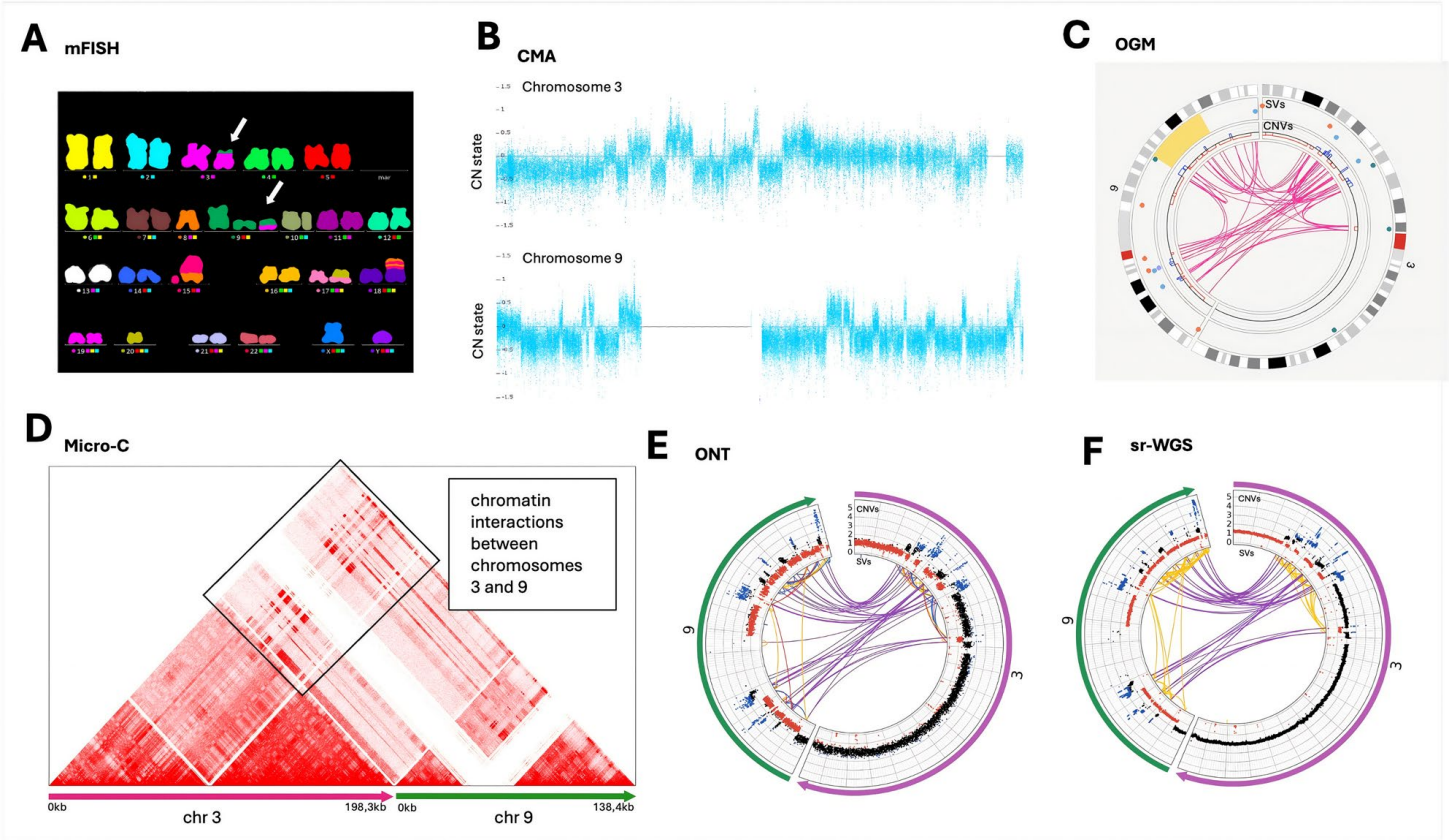
Demonstration of chromothripsis revealed in Pt 02. **A** MFISH initially identified a reciprocal translocation between chromosomes 3 and 9 *(der(3)t(3;9)(p?;?)* and *der(9)t(3;9)(p?;?)*, without any indications of chromothripsis. **B** However, further evaluation using CMA revealed CN oscillations on these chromosomes, a hallmark of chromothripsis. **C-F** The implementation of advanced genomic methods revealed the full complexity of these rearrangements. Advanced techniques uncovered a large number of SVs, including deletions, duplications, inversions, and translocations between chromosomes, alongside the observed CN oscillations. This case highlights the potential of advanced genomic approaches in uncovering complex genomic events that may be missed by traditional cytogenetic methods, underscoring their critical role in understanding the molecular basis of genomic instability.

Cth poses a significant interpretation challenge across genomic technologies due to its highly complex nature, characterized by clustered, localized chromosomal rearrangements and oscillating CN states. These features often evade detection or are misrepresented when analyzed using conventional approaches. In Pt. 02, CBA/mFISH reported a seemingly balanced TRA between chromosomes 3 and 9, annotated as SDER chr3 (*der(3)t(3;9)(p?;?))* and SDER chr9 (*der(9)t(3;9)(p?;?*)), in one of the clones (Fig. 7). However, CMA revealed extensive CN oscillations within the same region, a pattern highly suggestive of cth. This interpretation was further substantiated by ONT, OGM, sr-WGS, and Micro-C, which detected multiple SV types, including DELs, DUPs, INSs, INVs, and TRAs, that were co-localized within the affected genomic region.

**Fig. 8:**
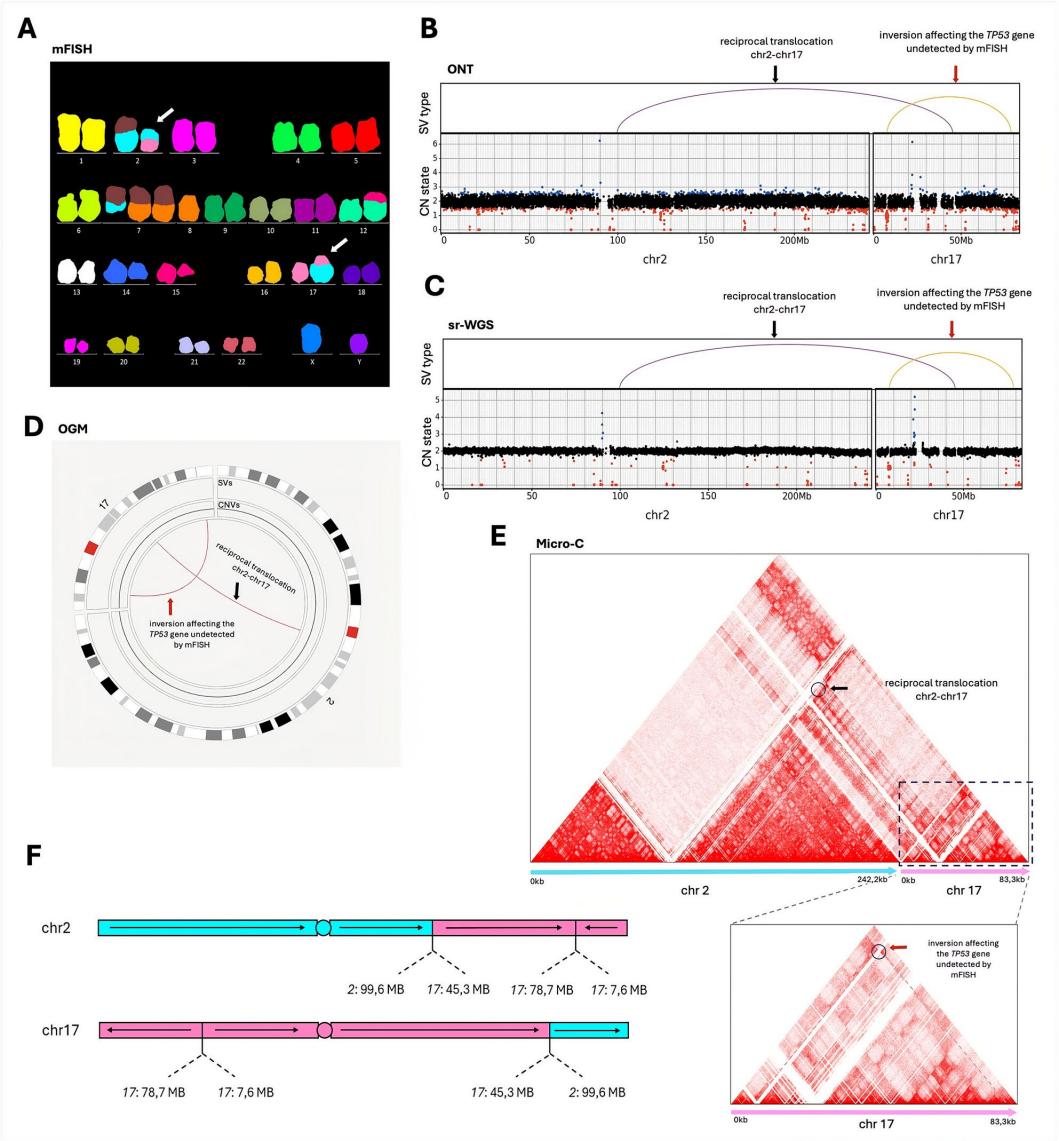
Resolved reciprocal translocation and detection of an inversion affecting the *TP53* gene, undetected by CBA/mFISH in Pt. 03. **A** CBA/mFISH analysis revealed two distinct translocation events involving chromosome 2: one chromosome 2 translocated with chromosome 17 *(t(2;17)(q?;q?))*, while the other with chromosome 7, with no additional aberrations observed on the affected chromosomes. **B-E** Advanced genomic methods confirmed the reciprocal translocation detected by CBA/mFISH (black arrow in the mFISH result). Notably, they also uncovered a large inversion of 71 Mb on chromosome 17, with BNDs located in the diagnostically important TP53 locus (red arrow). **F** A schematic presentation depicts the structural rearrangement RCP (2;17) with a co-occurring inversion on chromosome 17.

Additional examples illustrate how ONT, OGM, sr-WGS, and Micro-C provide critical insights beyond the scope of CBA/mFISH. In Pt. 03, an apparent RCP between chromosomes 2 and 17 (*t(2;17)(q?;q?)*) identified by CBA/mFISH was confirmed by all methods. However, further resolution uncovered a large INV on chromosome 17 spanning both arms and disrupting the *TP53* gene, a clinically relevant factor, an event undetected by CBA/mFISH (Fig. 8).

Similarly, in Pt. 09, CBA/mFISH identified two derivative chromosomes: SDER chr7 *der(7)t(7;8)(p?13;q?24*) and SDER chr9 *der(9)t(7;9)(p?13;q?33.1*) (Fig. 5). While these rearrangements implicated a TRA from chromosome 7 to chromosome 9 resulting in partial loss of chromosome 9, the precise genomic architecture remained unresolved. Integration of high-resolution methods revealed a preserved CN state two at the terminal region of chromosome 9, indicating that the missing segment was not lost but instead relocated. Subsequent analysis confirmed its fusion to the distal end of chromosome 8, in a region lacking CBA/mFISH signal following its TRA to chromosome 7.

These findings illustrate how low-resolution methods may oversimplify or incompletely resolve structurally complex events and underscore the necessity of high-resolution, integrative approaches for the accurate delineation not only of complex and cth-associated aberrations, but also of seemingly simple variants.

## Discussion

In this study, we provide the first comprehensive comparison of advanced high-throughput methods (ONT, sr-WGS, OGM, and Micro-C) in primary CLL samples with CKs. While previous comparative studies have mainly focused on cell lines from solid tumors or hematological malignancies [50–54], most often acute leukemias, no prior work has systematically evaluated these platforms alongside classical cytogenomic methods (CMA, CBA/mFISH) in CLL with CK. Despite the established clinical relevance of CK [5, 55, 56], the molecular mechanisms underlying its formation in CLL remain largely unexplored, primarily due to the limited resolution of conventional cytogenetic techniques. This gap highlights the need for integrative approaches capable of capturing the full spectrum and complexity of genomic aberrations in these high-risk cases.

Within our cohort, CMA demonstrated the highest sensitivity, specificity, and resolution for CNV detection, reaffirming its role as the clinical gold standard, while only sr-WGS achieved comparable performance. ONT, OGM, and Micro-C showed lower concordance for CNVs but proved more informative for SV detection. Across platforms, 10.9% of BNDs were consistently detected by all used methods, and 20.44% were supported by at least three methods. This limited overlap among the platforms may be attributed to factors such as sequencing depth, precision in breakpoint designation, and the reduced sensitivity for subclonal events, as noted in previous studies [50, 51]. These findings underscore that no single platform can fully capture CK complexity, emphasizing the importance of combining complementary methodologies.

Discrepancies across platforms reflect inherent differences in detection principles and analytical pipelines, including the availability of matched normal controls. Obtaining control cells from peripheral blood in high-risk leukemia patients may be problematic. Circulating blood may contain substantial proportions of leukemic cells and only limited numbers of normal cells, requiring laborious cell sorting, which is commonly not feasible for routine diagnostic use. Skin fibroblasts could be an alternative; however, obtaining them is challenging, and thus, other materials, such as buccal swabs that we used, are accepted in CLL [20], but typically yield relatively fragmented DNA. Consequently, matched normal material was available only for sr-WGS, enabling more accurate germline filtering. Matched controls were inaccessible for ONT and OGM in sufficient quality for HMW DNA isolation, or for Micro-C, which relies on viable cells from normal B-cell CLL counterparts rather than isolated DNA. Therefore, we decided to overcome these obstacles by filtering the ONT data against the 1000 Genomes dataset [36], which effectively removes common germline variants but may lack resolution for rare or cohort-specific variants.

Analytical settings also play a role, for instance, in Micro-C, the predefined bin size limits sensitivity for small SVs. Despite these limitations, Micro-C proved highly effective at confirming larger SVs, particularly TRAs, thereby complementing other platforms. This observation is consistent with previous reports, such as Mallard et al. [57], which demonstrated the utility of Hi-C in resolving clinically relevant TRAs, including the ETV6-RUNX1 translocation in pediatric B-ALL.

While there is growing enthusiasm for high-throughput technologies, their implementation in routine clinical practice remains constrained by technical demands, including complex bioinformatic analyses and high associated costs. Nonetheless, sr-WGS has been the primary sequencing-based method adopted in clinical workflows [58, 59]. Moreover, recent developments indicate that both ONT and OGM are increasingly being integrated into applied settings for specific use cases, supported by improvements in data quality and workflow standardization as reviewed previously [60, 61]. The added value of these platforms is further illustrated by comparing our BND dataset with the curated list of CLL-associated genes published by Knisbacher et al. [45]. Notably, in 5 of 9 patients, we identified SV BNDs affecting genes previously implicated in CLL pathogenesis, including *SPEN, SETD2, MGA, TP53, and GPS2.* These genes have been primarily associated with single-nucleotide or small indel mutations detected by gene sequencing (or deletions detected in the case of *SETD2*, *TP53, MGA*) [62–65]. Our findings suggest that SVs may also contribute to their disruption, potentially representing an alternative mechanism underlying disease progression in CK-CLL.

Among high-throughput technologies, OGM stands out as a particularly promising method for routine use, combining conceptual simplicity with a focus on cytogenetic-scale aberrations. Several studies suggested that OGM could serve as a viable alternative to multiple conventional assays, potentially consolidating cytogenomic diagnostics into a single comprehensive test [52, 53, 66]. Unlike sequencing-based methods, OGM is not designed to detect point mutations or small sequence variants, but it offers a graphical interface that enhances accessibility for laboratories that may lack extensive bioinformatic support, potentially facilitating broader adoption in routine cytogenomics. However, it is essential to consider the limitations of the method. In accordance with our findings, Shim et al. [67] reported a relatively low concordance rate in complex cases across various hematological malignancies when utilizing OGM. Nevertheless, despite these limitations, OGM’s ability to integrate multiple cytogenomic analyses into a single platform highlights its strong potential for practical and scalable clinical implementation.

Classical cytogenetics, particularly CBA/mFISH, remains essential for CK detection, providing indispensable context for complex structural phenomena. However, these methods are inherently limited by resolution, which depends on the quality of metaphase spreads derived from cultured cells. In tumor samples we analyzed, cytogenetic banding typically achieved a standardized resolution of 400 bands, which is insufficient to detect submicroscopic alterations or to define BNDs precisely. High-resolution platforms frequently revealed unexpected genomic complexity in regions previously interpreted as simple aberrations. This is exemplified by the detection of a clinically relevant pericentric INV of chromosome 17 in Pt. 03, where ONT, OGM, sr-WGS and Micro-C identified a BND disrupting the *TP53* gene, an aberration undetectable by CBA/mFISH and interphase FISH (Fig. 8). Similarly, in Pt. 09, a chromosomal imbalance involving a TRA between chromosomes 8 and 9 was missed by mFISH but accurately identified using high-resolution genomic approaches (Fig. 5). By contrast, CBA/mFISH maintained an advantage in detecting complex rearrangements such as DICs and CDERs that remain challenging to detect for sequencing-based approaches, particularly when breakpoints occur in repetitive or poorly mappable regions exceeding typical read lengths. The development of ultra-long ONT libraries offers the potential to resolve such rearrangements in the future, once these protocols become applicable for primary cancer samples with limited DNA input. Notably, Micro-C has proven particularly valuable in these cases, leveraging its ability to capture higher-order chromosomal interactions and architecture to bridge the gap between traditional cytogenetics and sequencing-based approaches. The implementation of complete human genome references, including also the T2T genome, may be critical for overcoming the limitations of sequencing-based approaches, offering significant potential for more accurate resolution of complex rearrangements in future applications, as illustrated by Pt. 04, in which a DIC involving chromosomes 11 and 18, initially identified by CBA/mFISH, was confirmed using Micro-C and further by ONT remapped to T2T (Fig. 6).

Overall, our findings indicate that in CK cases, the detection of CNVs, SVs, and specific chromosomal phenomena such as cth or DIC remains highly method-dependent, with no single platform providing a fully comprehensive and consistent profile (Table 2). Divergence between methods increases with genomic complexity, particularly in BND localization and variant classification. Although all technologies capture CK to some extent, given the limitations of each method, meaningful characterization of the underlying genomic landscape can only be achieved through a complementary approach. In this context, classical cytogenetic methods such as CBA/mFISH remain essential for detecting complex structural phenomena, while high-resolution genomic platforms enhance breakpoint resolution and reveal hidden layers of genomic complexity.

**Table 2:** Summary of the key features, applications, limitations, and added value of cytogenomic methods used for characterization of complex rearrangements

	CBA	mFISH	CMA	OGM	Sr-WGS	ONT	Micro-C
Material	chromosomes in the metaphase	chromosomes in the metaphase	DNA	UHMW-DNA	DNA	HMW-DNA	cross-linked DNA
Read length	NA	NA	NA	≥ 150 kb	300 bp	15–25 kb	300 bp
Throughput	+	+	+ +	+ + +	+ + +	+ + +	+ + +
Resolution	≥ 5–10 Mb	≥ 3–5 Mb	≥ 25 kb	≥ 500 bp	single nucleotide	single nucleotide	≥ 10 kb
Subclonal detection	+	+ + +	+ + +	+ +	+ +	+ +	+ +
Precise breakpoint detection	–	–	+ +	+ +	+ + +	+ + +	+
Unbalanced aberrations							
*DEL/DUP*	+	+	+ + +	+ + +	+ + +	+ + +	+ + +
*INS*	–	–	–	+ + +	–	+ + +	–
Balanced aberrations							
*INV*	+	+	–	+ + +	+ + +	+ + +	+ + +
*TRA*	+	+ +	–	+ + +	+ + +	+ + +	+ + +
Sensitivity for complex changes	+	+ +	+ +	+ + +	+ + +	+ + +	+ + +
Chromothripsis	–	–	+ +	+ + +	+ + +	+ + +	+ + +
Dicentric chromosomes	+ +	+ +	–	+ +	+ +	+ +	+ + +
Suitable for routine diagnostics	Yes	Yes	Yes	Increasingly	Yes	Increasingly	Research use only
Limitations	• Dependence on cell division • Low resolution	• Dependence on cell division • Low resolution	• Undetectable balanced rearrangements	• UHMW-DNA required • No information about sequence	• Poor mapping in repetitive regions	• HMW-DNA required • Bioinformatic challenges	• Indirect SVs detection • Standardized analytical pipelines missing
Added values	• Cost-effective and routinely used method	• Visualization of the whole karyotype	• High sensitivity for CNV detection	• Analytic software included	• Robust bioinformatic support and scalability	• Suitable for repetitive regions analysis	• 3D chromatin conformation

The table provides an overview of the key characteristics of employed technologies, focusing on their ability to detect SVs with respect to resolution, breakpoint precision, sensitivity to complex rearrangements, and applicability in clinical or research settings. Specific limitations associated with each method as well as unique advantages that may complement or surpass traditional techniques are highlighted. *UHMW-DNA–ultra-high molecular weight DNA; HMW-DNA–high-molecular weight DNA*

## Conclusion

Despite cytogenomic methods remaining the gold standard for CK-CLL diagnosis, many clinically significant variants likely remain undetected due to their limited resolution. Our study demonstrates that each high-throughput whole-genome analysis platform offers unique insights and can provide a comprehensive view of the CLL genome. By improving the detection of cryptic variants and uncovering additional structural alterations, these approaches have the potential to refine CK analysis, enhance the biological understanding of CLL pathogenesis, and support more precise patient risk stratification, although further validation in larger cohorts will be essential.

## Supplementary Information

Additional file 1: Supplementary_notes (.docx)—provides detailed additional information, such as extended methods, analyses, and data, that support and complement the main findings of the manuscript.

Additional file 2: Figures_supplementary (.pptx)—additional visual materials, such as graphs, plots, or images, that present supporting data or extended analyses not included in the main figures of the manuscript.

Additional file 3: SuppTab1_CBA and CMA records (.xlsx)—summary of CBA and CMA records

Additional file 4: SuppTab2_CNV_analysis (.xlsx)—comparison of CNV records across whole cohort and methods

Additional file 5: SuppTab3_BNDs_analysis (.xlsx)—comparison of BNDs records across whole cohort and methods

Additional file 6: SuppTab4_CBA_vs_advanced methods (.xlsx)—comparison of CBA records and advanced methods across whole cohort

Additional file 7: SuppTab5_CTH_BNDs (.xlsx)—comparison of BNDs associated with chromothripsis across whole cohort

## Data Availability

The research was conducted on primary samples of leukemia patients. Due to data protection policies, patient genomic sequences have not been submitted to publicly available databases. They will be made available upon adequate request and after institutional review.

## Abbreviations

AOH: Absence of Heterozygosity
BND: Breakend
CDER: Complex Derivative Chromosome
CK: Complex Karyotype
CLL: Chronic Lymphocytic Leukemia
CMA: Chromosomal Microarray
CNA: Copy Number Aberration
CNV: Copy Number Variation
cth: Chromothripsis
DEL: Deletion
DIC: Dicentric Chromosome
FISH: Fluorescence In Situ Hybridization
GC: Genomic Complexity
gDNA: Genomic DNA
Hi-C: High-throughput Chromosome Conformation Capture
HMW DNA: High-molecular Weight DNA
IGHV: Immunoglobulin Heavy-chain Variable Region
IGV: Integrative Genomics Viewer
INS: Insertion
INV: Inversion
ISCN: International System for Human Cytogenomic Nomenclature
LOH: Loss of Heterozygosity
LOSS: Chromosomal Loss
LOY: Loss of the Y Chromosome
mFISH: Multicolor Fluorescence In Situ Hybridization
NGS: Next-generation Sequencing
NOR: Nucleolus Organizer Regions
ONT: Oxford Nanopore Technologies; nanopore sequencing
RCP: Reciprocal Translocation
SDER: Simple Derivative Chromosome
Sr-WGS: Short-read Whole-genome Sequencing
SV: Structural Variant
*TP53*: Tumor Protein p53
UHMW DNA: Ultra-high-molecular Weight DNA
VAF: Variant Allele Frequency
