# Supplementary material for: Leveraging a spectrum of cytogenomics methods for profiling complex karyotypes in chronic lymphocytic leukemia
Source: Hum Genomics. 2026 Apr 11;20:88. doi: 10.1186/s40246-026-00957-4 (PMC13195900; doi:10.1186/s40246-026-00957-4)
Supplement: Supplementary file 1 — Additional file 1: Supplementary_notes (.docx)—provides detailed additional information, such as extended methods, analyses, and data, that support and complement the main findings of the manuscript. [file 40246_2026_957_MOESM1_ESM.docx]

# Supplementary notes

[1. Supplementary methods 2](#_Toc196746661)

[1.1 Methodological optimization of HMW DNA isolation and library preparation for ONT (Oxford Nanopore Technologies) long-read sequencing 2](#_Toc196746662)

[1.3 Bioinformatic analysis of long-read sequencing data (ONT) 3](#_Toc196746663)

[1.4 Laboratory workflow for Micro-C sample preparation 4](#_Toc196746664)

[1.5 Bioinformatic analysis of Micro-C data 4](#_Toc196746665)

[2. Parameters for SVs and CNVs Filtering 5](#_Toc196746666)

[2.1 Short-read sequencing 5](#_Toc196746667)

[2.2 Optical genome mapping 5](#_Toc196746668)

[3. Method comparison criteria 6](#_Toc196746669)

[3.1 CNV analysis 6](#_Toc196746670)

[3.2 BNDs analysis 8](#_Toc196746671)

[3.3 CBA/mFISH comparison 9](#_Toc196746672)

## 1. Supplementary methods

### **1.1** **Methodological optimization of HMW DNA isolation and library preparation for ONT (Oxford Nanopore Technologies) long-read sequencing**

High molecular weight DNA (HMW DNA) was isolated using chloroform-isopropanol extraction according to the following protocol: The cell pellet (3–50×10^6^) was resuspended in 1 ml of physiological saline, to which 3 ml of Fasano-A solution, consisting of 830 ml H_2_O, 40 ml 5M NaCl, 40 ml 0,5 EDTA, 40 ml 1M TRIS, 5 ml 10% SDS, and 20 µl of proteinase K at a concentration of 20 mg/ml (ZymoResearch, USA) were added. The mixture was incubated for 24 hours at 37°C. Following the incubation, 5.5 ml of chloroform and 1.4 ml of 5M NaCl were added. Continuous mixing was performed for 30 minutes. After centrifugation at 10,000 rpm for 10 minutes at 4°C, the aqueous phase containing HMW DNA was transferred to a clean 15 ml tube. An equal volume of room-temperature isopropanol was added. The precipitated HMW DNA was transferred to a 1.5 ml tube containing 200 µl of 70% ethanol. After centrifugation at 5400 rcf for 2 minutes, the supernatant was removed, and HMW DNA was dried at room temperature (10–15 minutes). HMW DNA was then dissolved in 400 µl TE with gentle shaking at 400 rpm for 24 hours at 37°C. The obtained HMW DNA was stored at 5°C. Before concentration measurement, samples were incubated for 20 minutes at 50°C and mixed thoroughly using a wide-bore pipette tip. 30G needles were used for fragmentation, aiming to obtain sequences of 15–20 kb in length. Short read elimination was performed by the Short Fragment Eliminator Expansion (ONT, United Kingdom) according to the manufacturer's instructions. To enhance yield, the initial reaction of HMW DNA with Short Fragment Eliminator Buffer (SFE) was scaled up to three times the manufacturer's recommended volume: 180 µl of HMW DNA at a concentration of 150 µl/ng and 180 µl of SFE buffer.

Four out of the nine samples were prepared using the Ligation Sequencing Kit SQK-LSK110 (ONT, United Kingdom). The remaining five were prepared using both SQK-LSK110 and SQK-LSK114. The input amount was increased to 5–10 µg. The subsequent sample processing with both kits followed the manufacturer's protocol, with the Hula mixer rotation extended to 15 minutes. SPRI magnetic beads (Beckman Coulter, USA) were used for sample purification. The sequencing library was eluted into a final volume of 51 µl.

For quality control, 1 µl (15–30 fmol) was loaded onto the Flongle Flow Cell. The initial five samples were sequenced on MinION Flow Cell R9.4.1, 100-200 fmol was loaded. Subsequent runs were initially performed using the PromethION Flow Cell R9.4.1. Following the release of the PromethION Flow Cell R10.4.1, the newer version was adopted for all later sequencing. In total, three samples were sequenced using a PromethION R9.4.1, three using a PromethION R10.4.1, and three samples were sequenced on both platforms. The PromethION R9.4.1 was loaded with 100–150 fmol, while the PromethION R10.4.1 was loaded with 60–80 fmol. Following a 24-hour sequencing period, the PromethION Flow Cell was washed in accordance with the protocol outlined in the Flow Cell Wash Kit (ONT, United Kingdom). Typically, two to three portions of the library were loaded, based on sequencing efficiency and the available library amount.

### **1.3** **Bioinformatic analysis of long-read sequencing data (ONT)**

To enhance data consistency, data obtained from different sequencing devices were merged at the initial stage of bioinformatic analysis. Basecalling was performed with models specific to the sequencing chemistry. For V14 chemistry, the dna_r10.4.1_e8.2_400bps_sup@v4.3.0 model was employed, while the dna_r9.4.1_e8_sup@v3.6 model was used for V10 chemistry. The basecalling process generated FASTQ files, which were subsequently aligned to the GRCh38 reference genome using Minimap2 (v2.26-r1175) in map-ont mode ([PMC6137996](https://pubmed.ncbi.nlm.nih.gov/29750242/)). This mode is specifically optimized to accommodate the unique properties of nanopore sequencing reads. Additionally, some analyses were performed using the remapped reads to the T2T-CHM13 reference genome.

### **1.4** **Laboratory workflow for Micro-C sample preparation**

The Micro-C libraries were prepared using the Dovetail® Micro-C Kit (protocol versions 1.2 or 2.0, available on <https://cantatabio.com/wp-content/uploads/2023/12/Dovetail%C2%AE-Micro-C-Kit-User-Guide-Version-1.2.pdf>; <https://cantatabio.com/wp-content/uploads/2024/05/Dovetail%C2%AE-Micro-C-Kit-User-Guide-Version-2_May2024.pdf>), following the manufacturer’s instructions. The protocol was modified by increasing the input to 2 million cells and by optimizing the enzyme concentration in stage 1 for each sample based on quality control and fragmentation profiles at the end of stage 2. Briefly, chromatin was fixed with disuccinimidyl glutarate (DSG) and formaldehyde in the nucleus. The cross-linked chromatin was digested *in situ* with micrococcal nuclease (*MNase*). Following digestion, the cells were lysed with SDS to extract the chromatin fragments, and the chromatin fragments were bound to Chromatin Capture Beads. Next, the chromatin ends were repaired and ligated to a biotinylated bridge adapter, followed by proximity ligation of adapter-containing ends. After proximity ligation, the crosslinks were reversed, the associated proteins were degraded, and the DNA was purified and converted into a sequencing library using Illumina-compatible adaptors and Ultra II DNA Library Prep Kit for Illumina (New England Biolabs, USA). Biotin-containing fragments were isolated using streptavidin beads prior to PCR amplification.

### **1.5** **Bioinformatic analysis of Micro-C data**

Paired-end reads were aligned to the hg38 reference genome using the BWA-MEM algorithm. For samples with technical replicates (three out of nine samples from the cohort), FASTQ files were merged before alignment. Reads with low mapping quality (MAPQ <40) or unmapped reads were excluded. *Pairtools* was used to flag and remove PCR duplicates and to categorize non-duplicate, high-quality read pairs (MAPQ ≥40) as valid if: a) the reads mapped to different chromosomes (*trans*), or b) to the same chromosome (*cis*) with a genomic distance > 1 kb ([PMC11164360](https://pmc.ncbi.nlm.nih.gov/articles/PMC11164360/)).

## 2. Parameters for SVs and CNVs Filtering

This section outlines the filtering strategies implemented for the structural variant (SV) and copy number variant (CNV) datasets derived from advanced genomic technologies. Stringent thresholds and standardized criteria were applied to optimize data quality, maximize analytical robustness, and ensure comparability across all downstream analyses.

### **2.1** **Short-read sequencing**

Several filtering steps were applied to distinguish true somatic variants from germline or artifact calls. Pre-filtered SVs were genotyped, where candidate sites were re-evaluated across multiple control samples. This additional genotyping step was crucial in improving the robustness and accuracy of the somatic SV calls, ensuring consistent detection across samples. For downstream analysis, SVs smaller than 1,000 bp were filtered out and not considered further.

### **2.2** **Optical genome mapping**

For each sample, the following embedded filtering criteria were used: For SVs, confidence scores were set as recommended, and masking, size, and VAF filters were set to be fully inclusive. To exclude common germline SVs, all the SVs present in the Control database (285 individuals) in >1% were filtered out. The remaining SV filtering criteria were left in the default state. For CNV detection, default filtering criteria were applied.

All SVs and CNVs records were manually curated and filtered: Considering CNVs, each record was checked in Access software in Whole Genome view, and adjacent segments with similar CN state (difference < 0.5) were manually joined into one record. The incorrectly assigned type of missing gonosome (X vs. Y) in a record was corrected with the knowledge of the patient’s sex and visual inspection of the copy number plot in Access. As for SVs, each record was checked in Access in Genome Browser for its reliability (e.g., presence of molecules spanning both sides of the breakend (BND), a sufficient number of fitting labels on both sides, presence of alternative alignments). Unreliable records and redundant records describing the same SV were eliminated. In some cases, the type of SV was corrected, considering the surrounding genome map and CN state.

## 3. Criteria for method comparison

### **3.1** **CNV analysis**

CNV calls from advanced methods were compared to CMA-detected CN changes based on two primary criteria: (1) at least one BND (either start or end) fell within a distance range, as defined in the following text, and (2) the CNV spanned at least 80% of the corresponding CMA-detected region. The BND distance tolerance varied depending on the resolution of each method. For instance, sr-WGS, with the segmentation of a 10kb interval from the Delly pipeline, displayed high precision, with BND typically deviating within 10kb from CMA. Micro-C, analyzed with a bin size of 25kb, exhibited BND deviations up to 50kb, reflecting its lower resolution. OGM and ONT, due to their inherent resolution limits in CNV analysis, showed fewer BNDs, with deviations around 50 kb. ONT demonstrated the highest variability, often displaying BND up to 100 kb from CMA, due to probable uneven coverage and CNV detection divergence because of missing matching normal samples to tumor samples. For CNVs located in repetitive regions (e.g., centromeres, telomeres, and acrocentric regions), BND discrepancies were observed across methods, likely due to challenges in mapping and segmentations in these regions. In such cases, regional overlap rather than precise BND alignment was used to determine concordance, as the resolution limitations of different approaches rendered exact BND localization unreliable.

To further standardize CNV calls across methods, we implemented a merging approach to unify fragmented CNVs. Given the higher resolution and more established analytical frameworks of sr-WGS (which included tumor-normal matching samples) and CMA, these methods often detected multiple adjacent CNVs. In contrast, lower-resolution methods (OGM, ONT, and Micro-C) identified a single broader event encompassing the same region. In such cases, CNV records were merged into a single event labeled as “detected by all methods” if the type of alteration was consistent across methods (e.g., all methods consistently detected only losses or gains). However, if CMA or sr-WGS identified additional subregions (e.g., a small gain embedded within a larger loss or conversely) that were below the detection limit of other methods, these were annotated separately (e.g., “CMA or sr-WGS only”).

The dataset was further filtered to exclude BND overlapping immunoglobulin (IG) loci, specifically the IG kappa light chain locus (IGK; chr2:88,866,370–90,790,947), the IG heavy chain locus (IGH; chr14:105,586,437–106,879,843), and the IG lambda light chain locus (IGL; chr22:22,439,629–23,345,823), resembling physiologically programmed B-cell receptor rearrangements during B-cell maturation.

### **3.2** **BNDs analysis**

Correspondingly oriented BNDs from SVs and CNVs that were on the same chromosome and were less than 100 kb apart were merged into a single record, as well as BNDs where the CNV state changed at the same position (e.g., loss to gain and vice versa). For merging BNDs from CNVs, the tolerance was extended up to 1 Mb. However, in regions with multiple BND clusters, often indicative of chromothripsis, these BNDs were not merged to preserve the characteristic structural pattern. During the comparison process, BNDs were evaluated based on the distance between BNDs detected by each method, with a threshold of <100 kb, as well as their directionality: if the direction was consistent across all methods, the BNDs were considered the same. However, discrepancies were observed in some methods, where the directions and SV types associated with the BND did not align across all methods. These discrepancies were particularly common in complex or cth regions, where multiple rearrangements occurred within a single chromosomal segment. Similarly to CNV analysis, BNDs located within immunoglobulin loci (IGH, IGK, and IGL) were excluded from the final dataset.

### **3.3** **CBA/mFISH comparison**

BNDs identified by advanced methods were systematically mapped to cytoband coordinates to allow for direct comparison with cytogenetic annotations. Given the limitations of cytogenetic resolution, minor discrepancies (i.e., adjacent cytobands) were tolerated, whereas more significant differences (i.e., between chromosome arms) were evaluated individually in the context of whole complex rearrangement. In such cases, advanced methods provided crucial contextual information, enabling refined interpretation beyond the scope of conventional cytogenetics. We used a three-tier classification based on three scenarios: the cytogenetic record was a) confirmed (“yes”), b) not detected (“no”), or c) partially confirmed (“partly”). The latter case applied primarily to complex rearrangements, where only a part of all BNDs included in the CBA record were confirmed by advanced methods (Fig. 5B).
