# Supplementary material for: Leveraging a spectrum of cytogenomics methods for profiling complex karyotypes in chronic lymphocytic leukemia
Source: Hum Genomics. 2026 Apr 11;20:88. doi: 10.1186/s40246-026-00957-4 (PMC13195900; doi:10.1186/s40246-026-00957-4)
Supplement: Supplementary file 2 — Additional file 2: Figures_supplementary (.pptx)—additional visual materials, such as graphs, plots, or images, that present supporting data or extended analyses not included in the main figures of the manuscript. [file 40246_2026_957_MOESM2_ESM.pptx]

## Slide 1
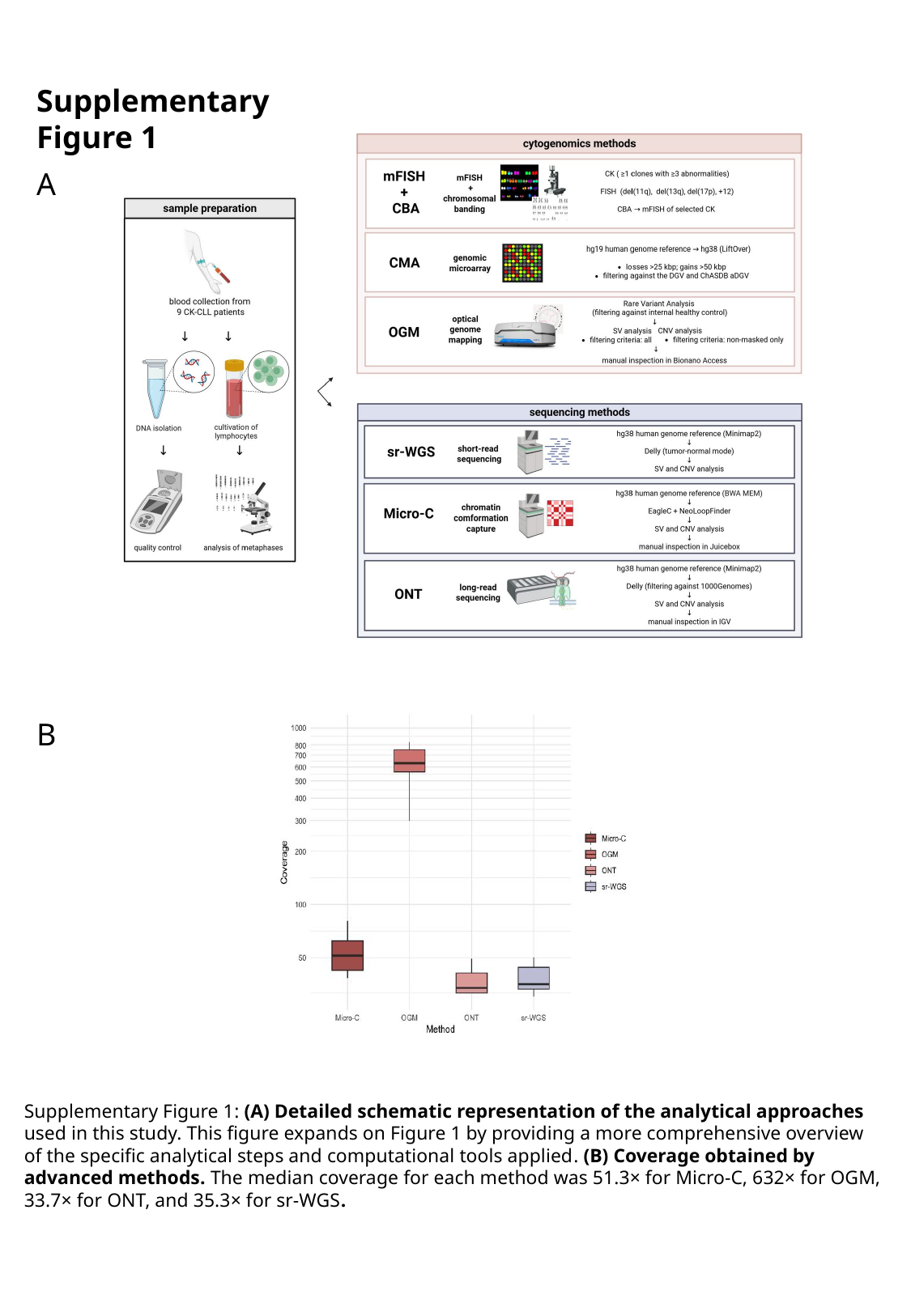

Supplementary Figure 1
A
B
Supplementary Figure 1: (A) Detailed schematic representation of the analytical approaches used in this study. This figure expands on Figure 1 by providing a more comprehensive overview of the specific analytical steps and computational tools applied. (B) Coverage obtained by advanced methods. The median coverage for each method was 51.3× for Micro-C, 632× for OGM, 33.7× for ONT, and 35.3× for sr-WGS.

## Slide 2
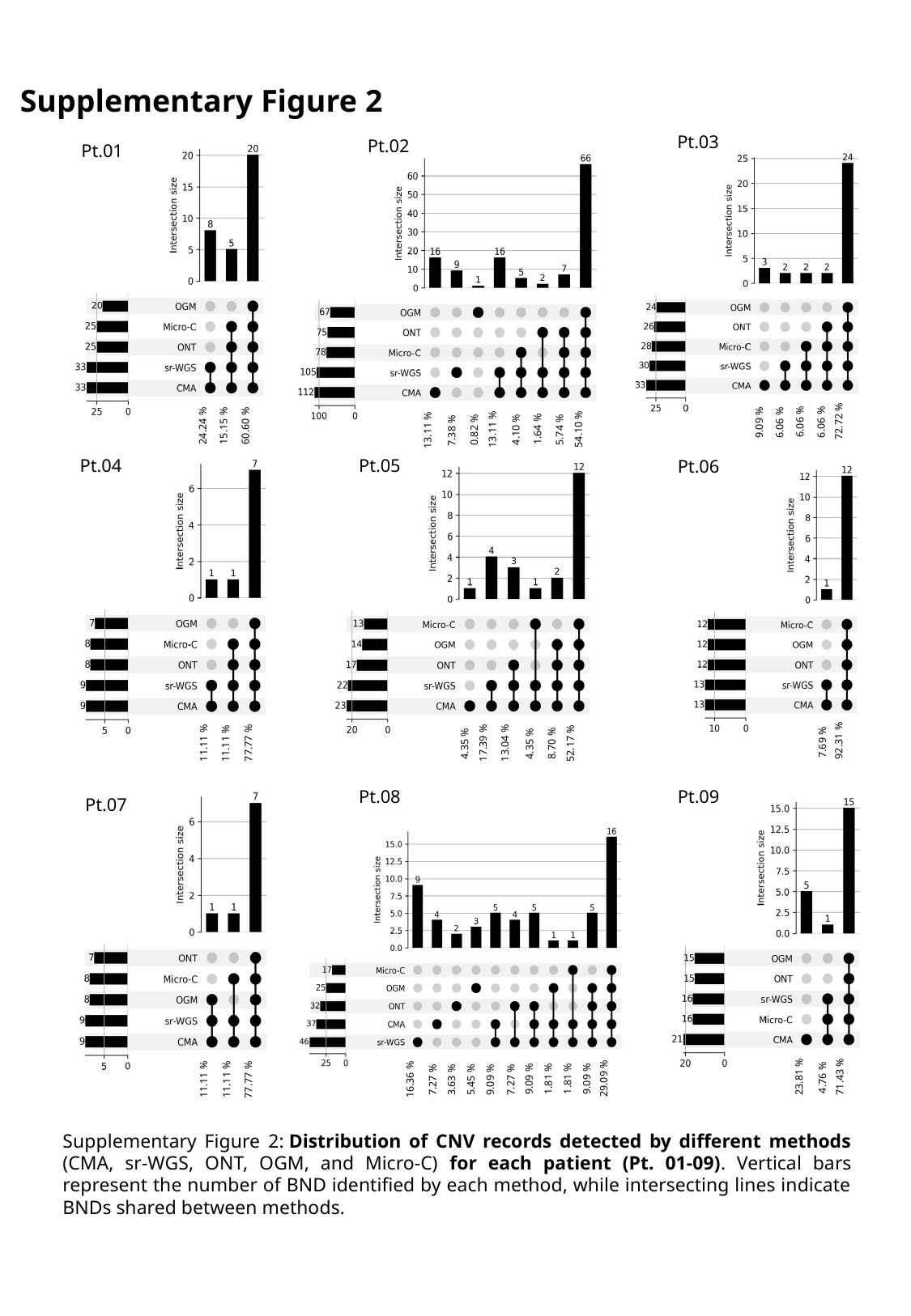

Supplementary Figure 2
Pt.03
Pt.02
1.64 %
4.10 %
0.82 %
7.38 %
13.11 %
13.11 %
5.74 %
54.10 %
Pt.01
24.24 %
15.15 %
60.60 %
6.06 %
9.09 %
6.06 %
6.06 %
72.72 %
Pt.05
Pt.04
Pt.06
8.70 %
4.35 %
4.35 %
13.04 %
17.39 %
52.17 %
7.69 %
92.31 %
77.77 %
11.11 %
11.11 %
Pt.08
Pt.09
11.11 %
11.11 %
77.77 %
Pt.07
4.76 %
23.81 %
71.43 %
1.81 %
1.81 %
9.09 %
9.09 %
7.27 %
9.09 %
5.45 %
7.27 %
3.63 %
29.09 %
16.36 %
Supplementary Figure 2: Distribution of CNV records detected by different methods (CMA, sr-WGS, ONT, OGM, and Micro-C) for each patient (Pt. 01-09). Vertical bars represent the number of BND identified by each method, while intersecting lines indicate BNDs shared between methods.

## Slide 3
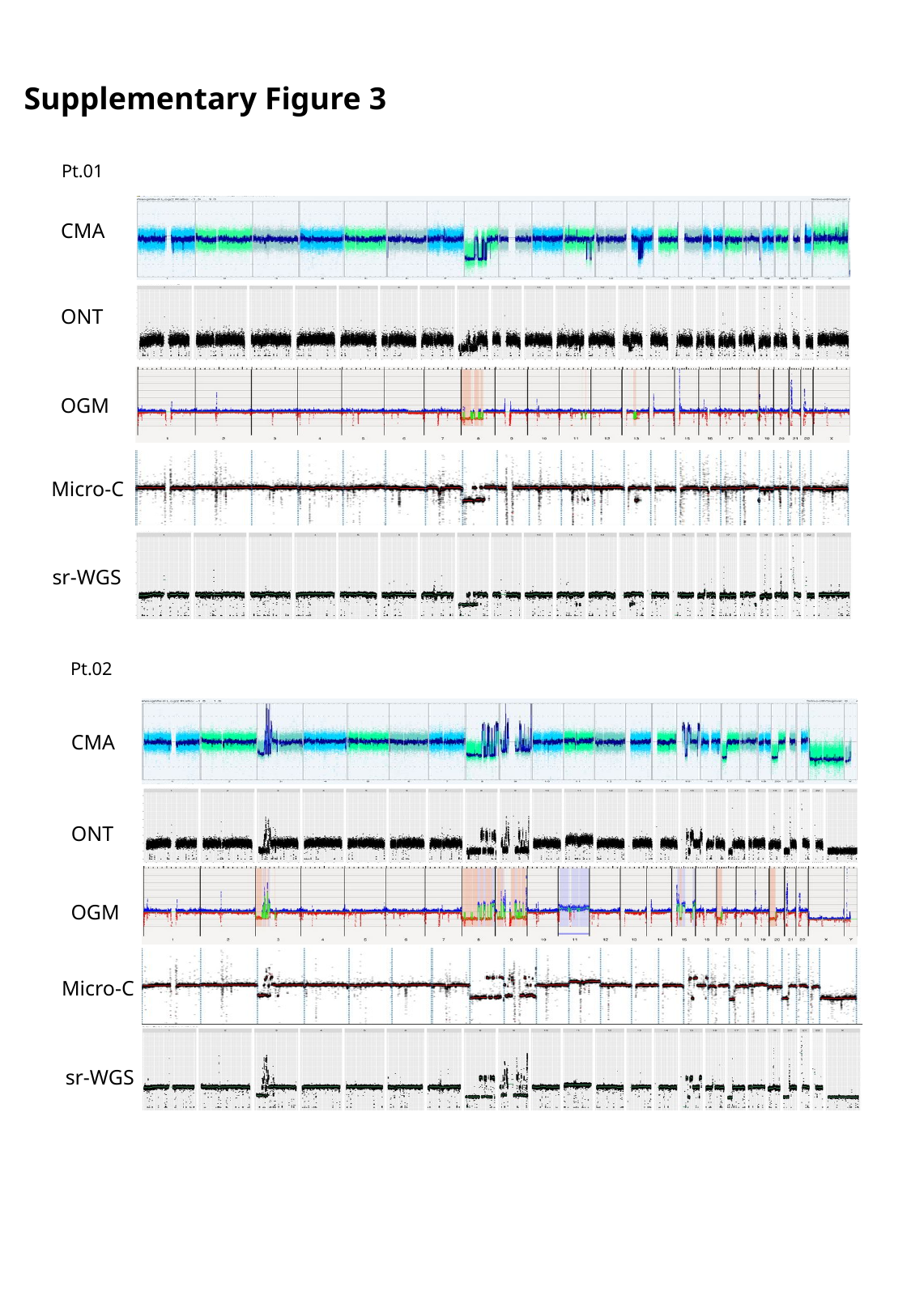

Supplementary Figure 3
Pt.01
CMA
ONT
OGM
Micro-C
sr-WGS
Pt.02
CMA
ONT
OGM
Micro-C
sr-WGS

## Slide 4
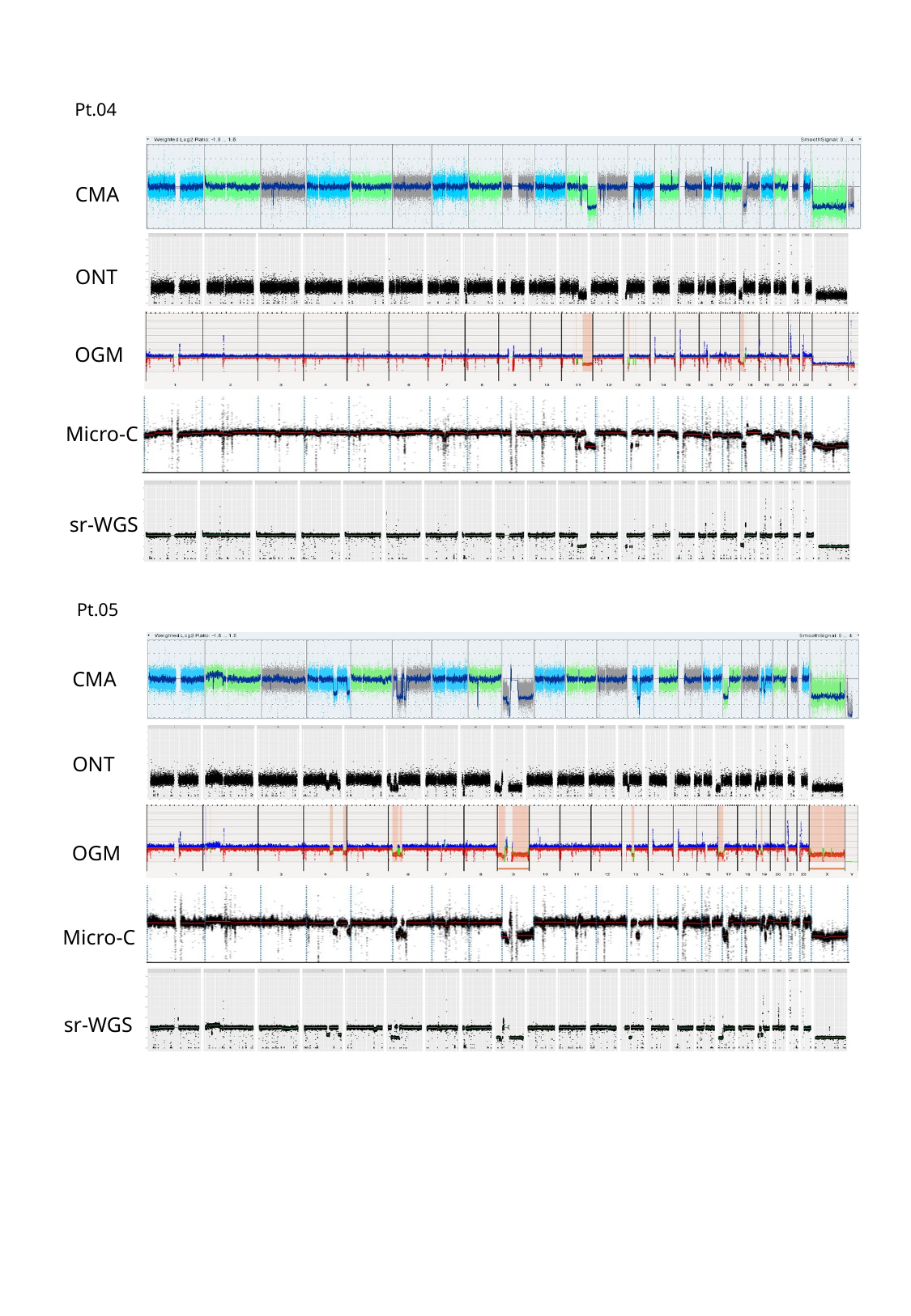

Pt.04
CMA
ONT
OGM
Micro-C
sr-WGS
Pt.05
CMA
ONT
OGM
Micro-C
sr-WGS

## Slide 5
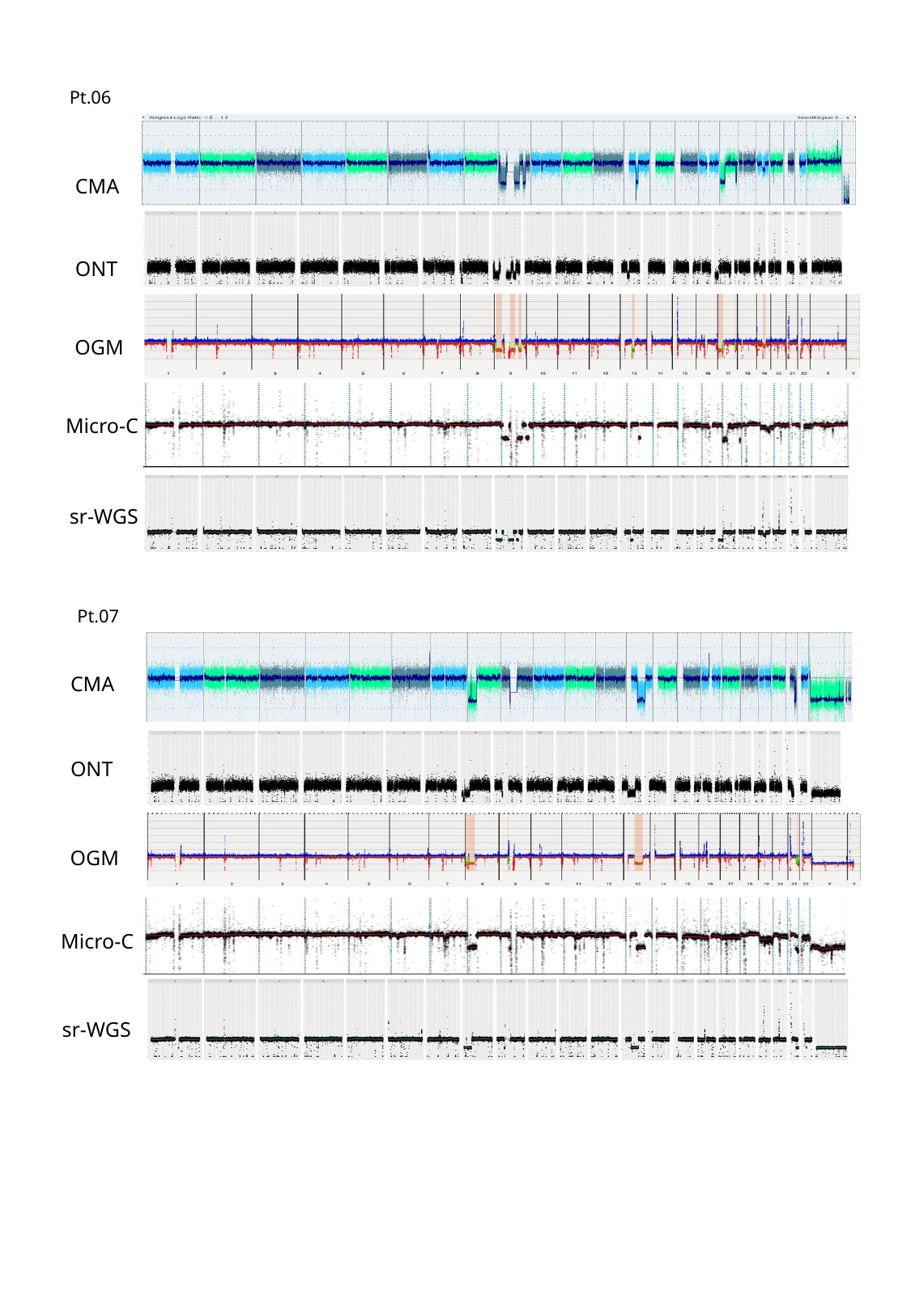

Pt.06
CMA
ONT
OGM
Micro-C
sr-WGS
Pt.07
CMA
ONT
OGM
Micro-C
sr-WGS

## Slide 6
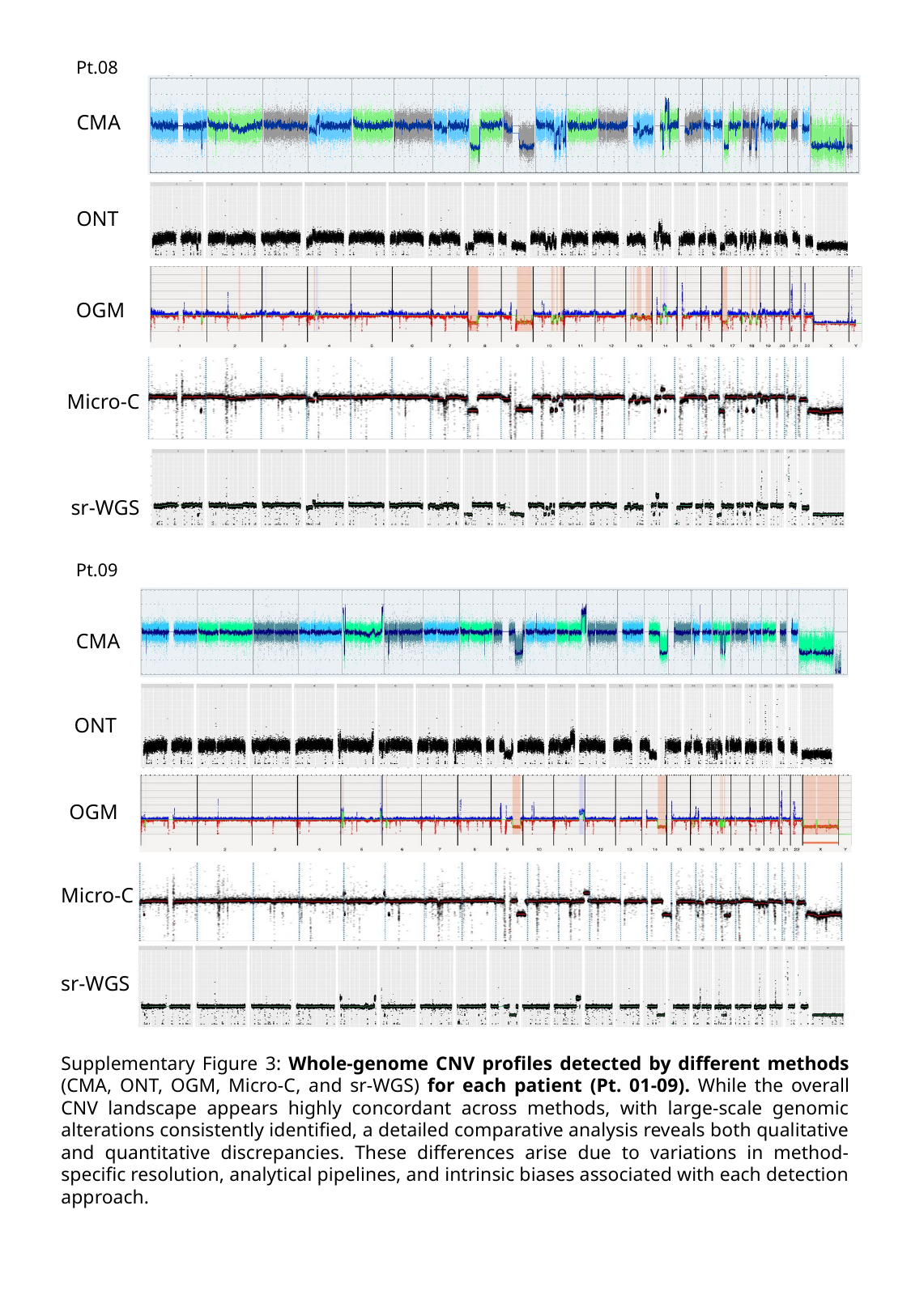

Pt.08
CMA
ONT
OGM
Micro-C
sr-WGS
Pt.09
CMA
ONT
OGM
Micro-C
sr-WGS
Supplementary Figure 3: Whole-genome CNV profiles detected by different methods (CMA, ONT, OGM, Micro-C, and sr-WGS) for each patient (Pt. 01-09). While the overall CNV landscape appears highly concordant across methods, with large-scale genomic alterations consistently identified, a detailed comparative analysis reveals both qualitative and quantitative discrepancies. These differences arise due to variations in method-specific resolution, analytical pipelines, and intrinsic biases associated with each detection approach.

## Slide 7
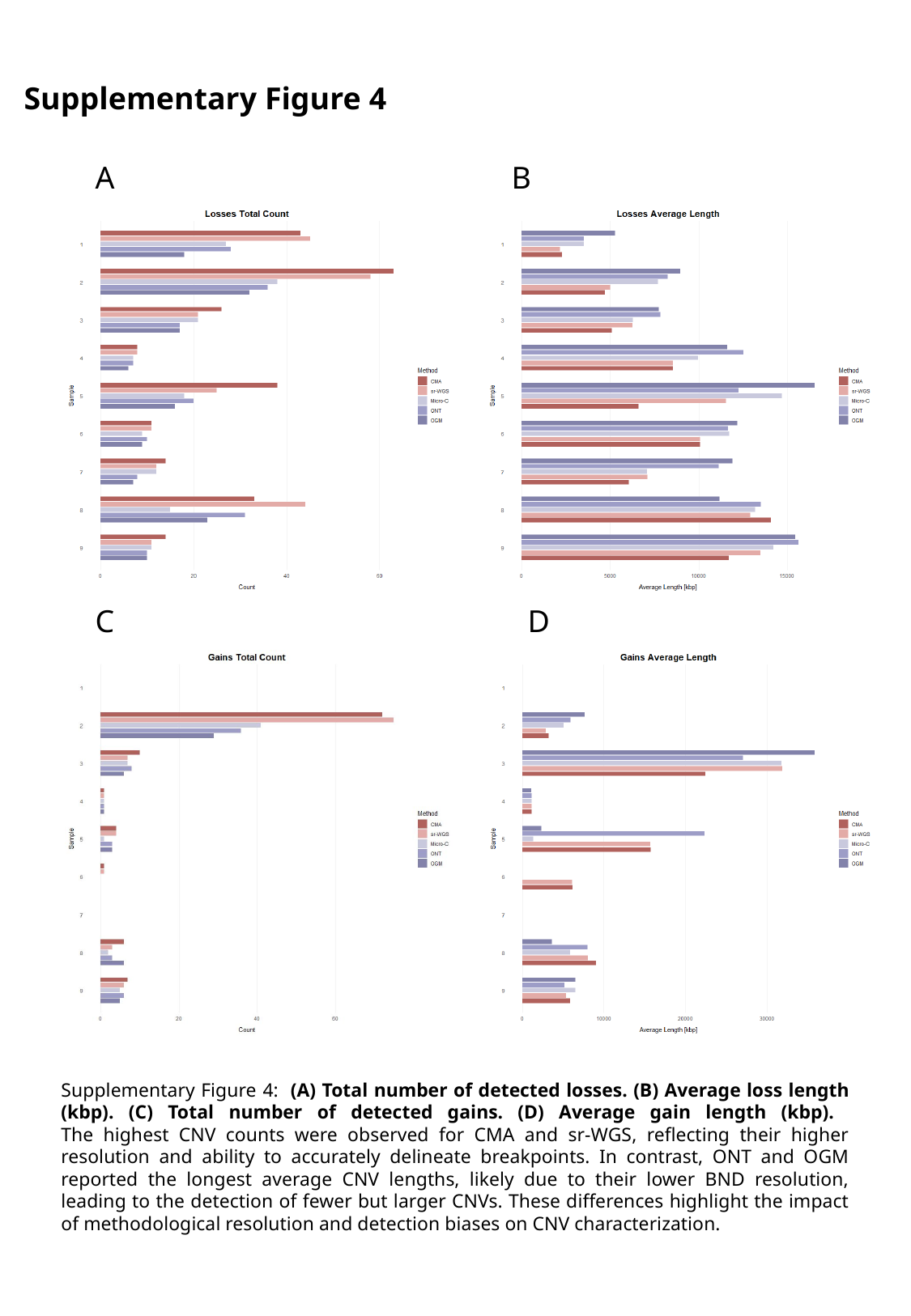

Supplementary Figure 4
A
B
C
D
Supplementary Figure 4: (A) Total number of detected losses. (B) Average loss length (kbp). (C) Total number of detected gains. (D) Average gain length (kbp).
The highest CNV counts were observed for CMA and sr-WGS, reflecting their higher resolution and ability to accurately delineate breakpoints. In contrast, ONT and OGM reported the longest average CNV lengths, likely due to their lower BND resolution, leading to the detection of fewer but larger CNVs. These differences highlight the impact of methodological resolution and detection biases on CNV characterization.

## Slide 8
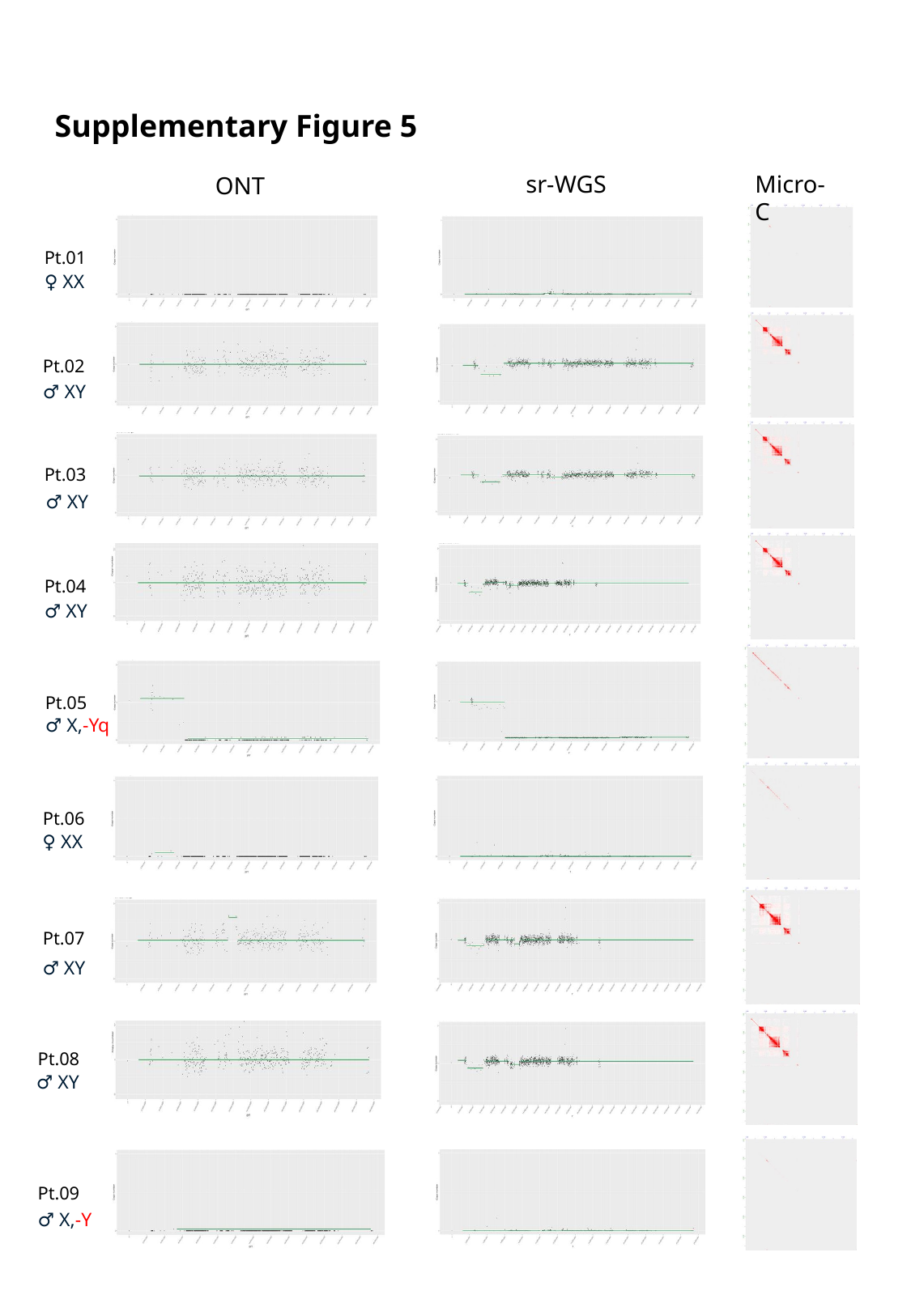

Supplementary Figure 5
Micro-C
sr-WGS
ONT
Pt.01
♀ XX
Pt.02
♂ XY
Pt.03
♂ XY
Pt.04
♂ XY
Pt.05
♂ X,-Yq
Pt.06
♀ XX
Pt.07
♂ XY
Pt.08
♂ XY
Pt.09
♂ X,-Y

## Slide 9
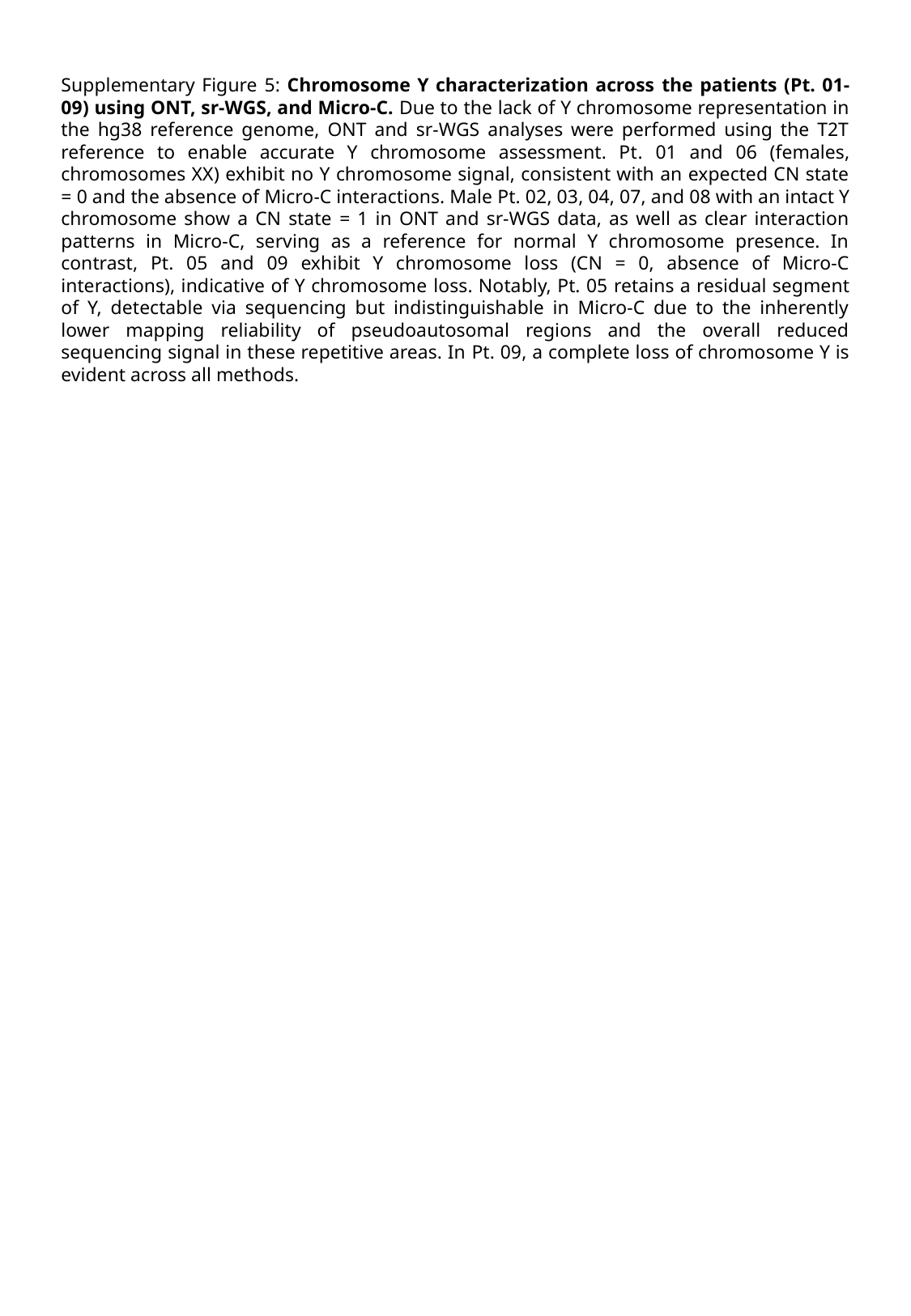

Supplementary Figure 5: Chromosome Y characterization across the patients (Pt. 01-09) using ONT, sr-WGS, and Micro-C. Due to the lack of Y chromosome representation in the hg38 reference genome, ONT and sr-WGS analyses were performed using the T2T reference to enable accurate Y chromosome assessment. Pt. 01 and 06 (females, chromosomes XX) exhibit no Y chromosome signal, consistent with an expected CN state = 0 and the absence of Micro-C interactions. Male Pt. 02, 03, 04, 07, and 08 with an intact Y chromosome show a CN state = 1 in ONT and sr-WGS data, as well as clear interaction patterns in Micro-C, serving as a reference for normal Y chromosome presence. In contrast, Pt. 05 and 09 exhibit Y chromosome loss (CN = 0, absence of Micro-C interactions), indicative of Y chromosome loss. Notably, Pt. 05 retains a residual segment of Y, detectable via sequencing but indistinguishable in Micro-C due to the inherently lower mapping reliability of pseudoautosomal regions and the overall reduced sequencing signal in these repetitive areas. In Pt. 09, a complete loss of chromosome Y is evident across all methods.

## Slide 10
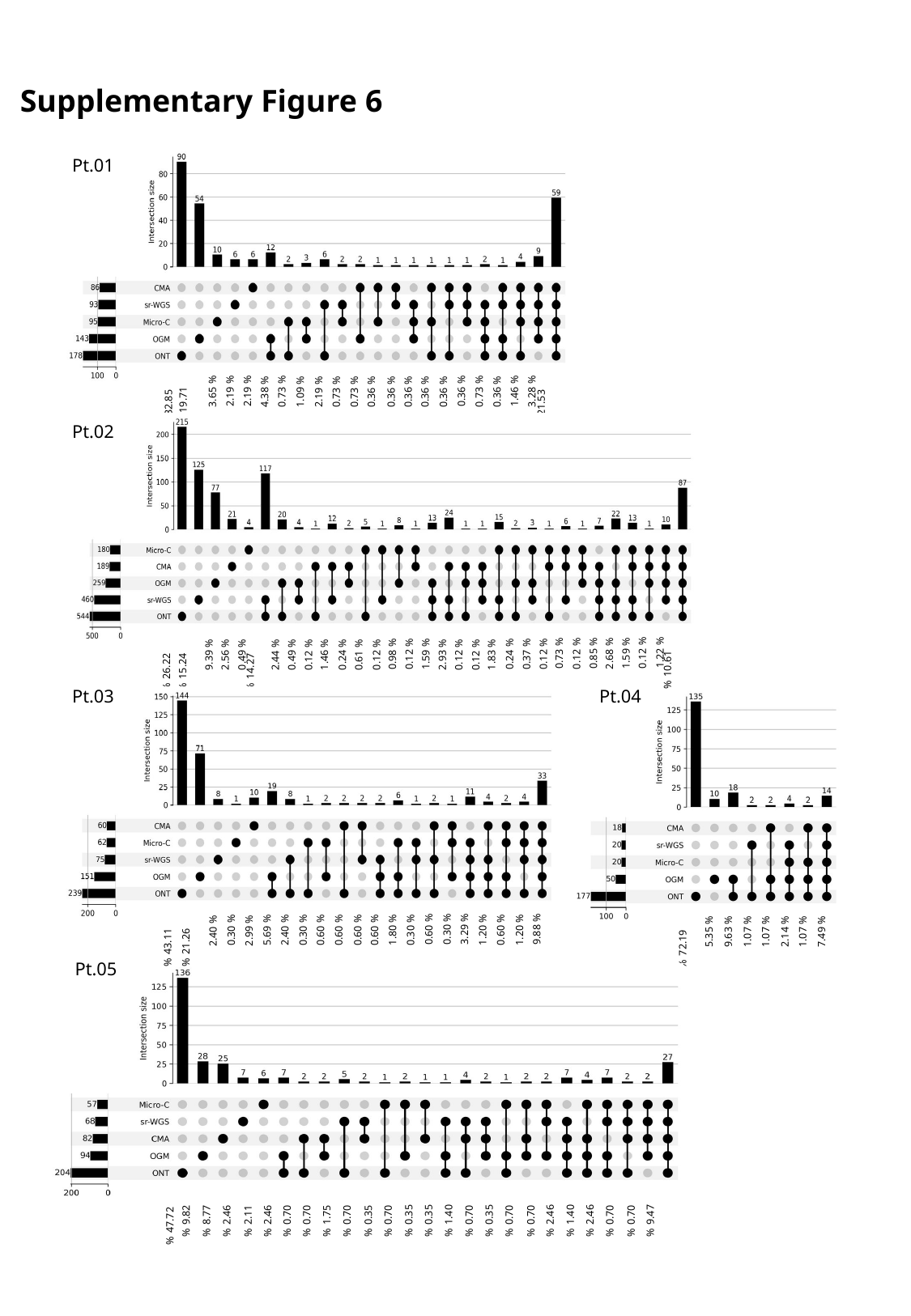

Supplementary Figure 6
Pt.01
2.19 %
2.19 %
1.46 %
0.36 %
3.65 %
0.73 %
0.73 %
3.28 %
1.09 %
0.36 %
0.36 %
4.38 %
2.19 %
0.73 %
0.73 %
0.36 %
0.36 %
0.36 %
0.36 %
32.85 %
19.71 %
21.53 %
1.22 %
0.12 %
0.85 %
1.59 %
0.73 %
2.68 %
0.12 %
0.12 %
0.12 %
0.98 %
0.24 %
0.37 %
1.59 %
0.49 %
1.46 %
0.12 %
0.24 %
1.83 %
2.56 %
0.12 %
2.93 %
9.39 %
0.49 %
0.61 %
0.12 %
0.12 %
10.61 %
2.44 %
15.24 %
14.27 %
26.22 %
Pt.02
Pt.03
Pt.04
0.30 %
3.29 %
9.88 %
0.60 %
1.80 %
1.20 %
0.60 %
1.20 %
0.30 %
0.60 %
0.30 %
5.69 %
2.40 %
0.30 %
0.60 %
0.60 %
0.60 %
2.99 %
2.40 %
43.11 %
21.26 %
5.35 %
9.63 %
1.07 %
1.07 %
2.14 %
72.19 %
1.07 %
7.49 %
Pt.05
0.35 %
0.35 %
1.40 %
0.35 %
2.46 %
1.40 %
2.46 %
9.47 %
0.70 %
0.35 %
9.82 %
8.77 %
2.46 %
2.11 %
2.46 %
0.70 %
0.70 %
1.75 %
0.70 %
0.70 %
0.70 %
0.70 %
0.70 %
0.70 %
47.72 %

## Slide 11
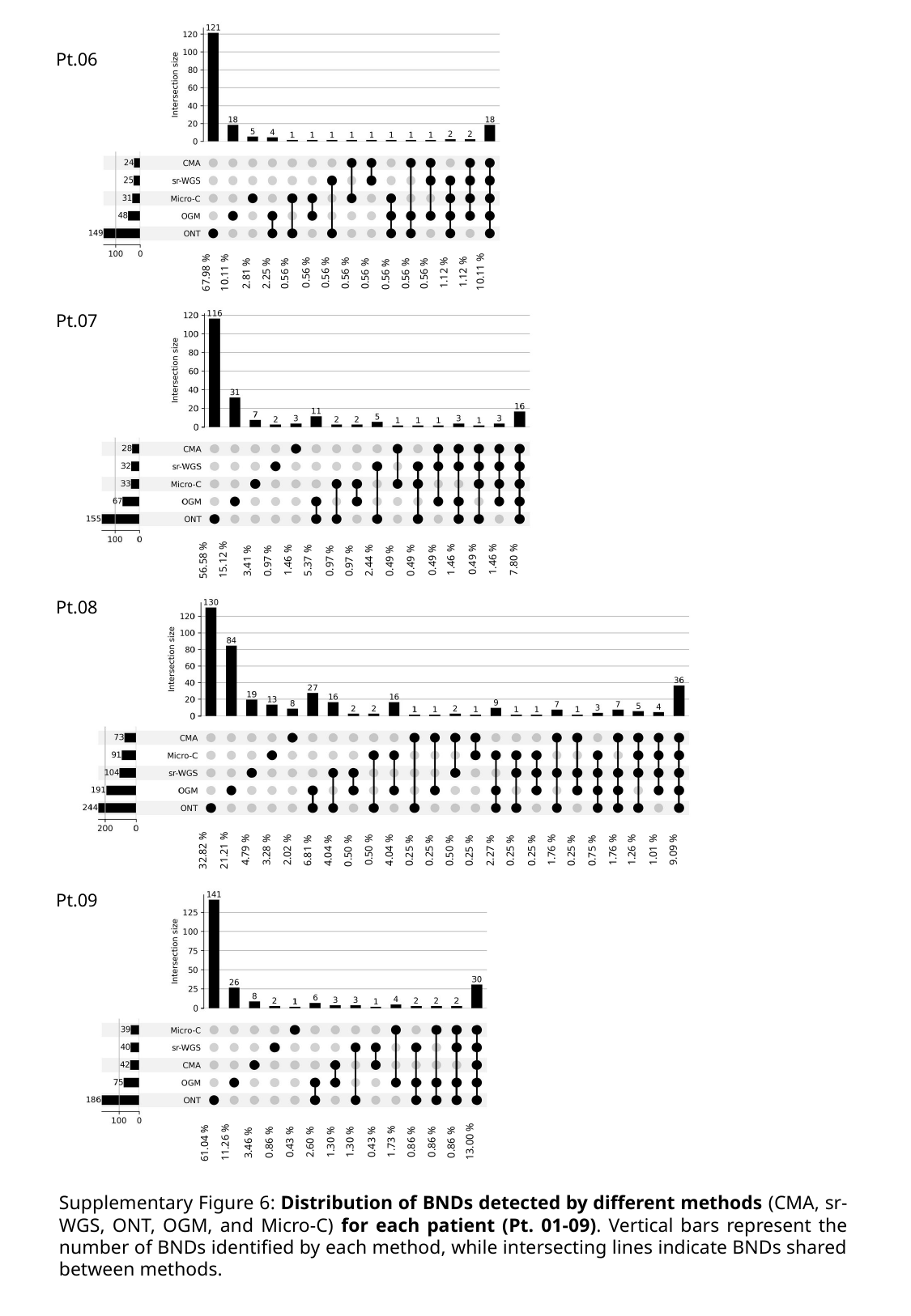

1.12 %
0.56 %
1.12 %
0.56 %
0.56 %
2.81 %
2.25 %
0.56 %
0.56 %
0.56 %
0.56 %
0.56 %
10.11 %
67.98 %
10.11 %
Pt.06
1.46 %
0.49 %
1.46 %
7.80 %
0.49 %
1.46 %
2.44 %
5.37 %
0.49 %
0.97 %
0.97 %
0.49 %
3.41 %
0.97 %
15.12 %
56.58 %
Pt.07
Pt.08
9.09 %
0.50 %
1.76 %
1.76 %
1.26 %
1.01 %
2.02 %
4.79 %
3.28 %
4.04 %
0.25 %
2.27 %
0.25 %
0.25 %
0.25 %
6.81 %
4.04 %
0.25 %
0.50 %
0.75 %
0.25 %
0.50 %
32.82 %
21.21 %
Pt.09
1.73 %
0.86 %
1.30 %
1.30 %
0.43 %
0.86 %
0.43 %
2.60 %
0.86 %
0.86 %
3.46 %
13.00 %
11.26 %
61.04 %
Supplementary Figure 6: Distribution of BNDs detected by different methods (CMA, sr-WGS, ONT, OGM, and Micro-C) for each patient (Pt. 01-09). Vertical bars represent the number of BNDs identified by each method, while intersecting lines indicate BNDs shared between methods.

## Slide 12
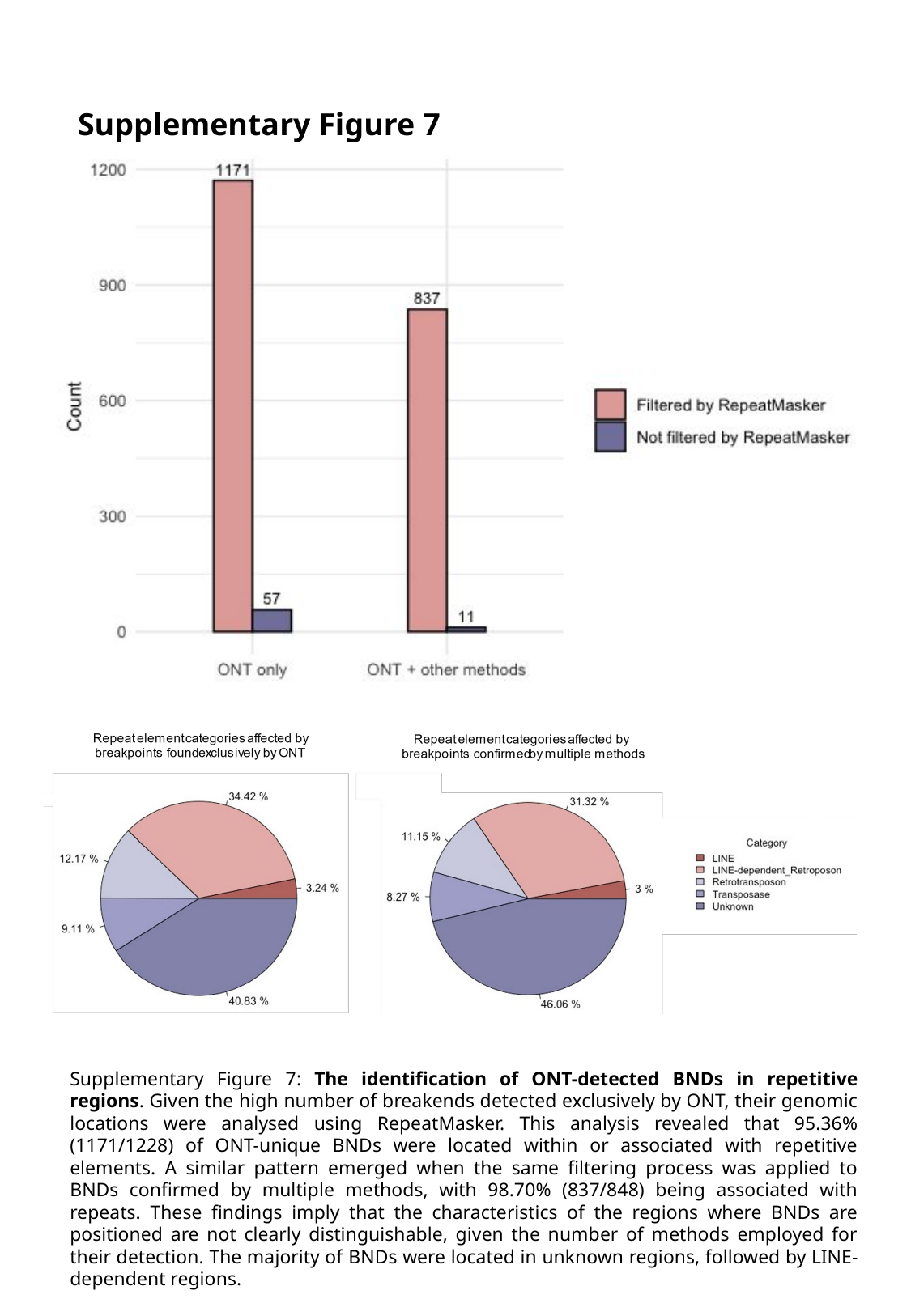

Supplementary Figure 7
Supplementary Figure 7: The identification of ONT-detected BNDs in repetitive regions. Given the high number of breakends detected exclusively by ONT, their genomic locations were analysed using RepeatMasker. This analysis revealed that 95.36% (1171/1228) of ONT-unique BNDs were located within or associated with repetitive elements. A similar pattern emerged when the same filtering process was applied to BNDs confirmed by multiple methods, with 98.70% (837/848) being associated with repeats. These findings imply that the characteristics of the regions where BNDs are positioned are not clearly distinguishable, given the number of methods employed for their detection. The majority of BNDs were located in unknown regions, followed by LINE-dependent regions.

## Slide 13
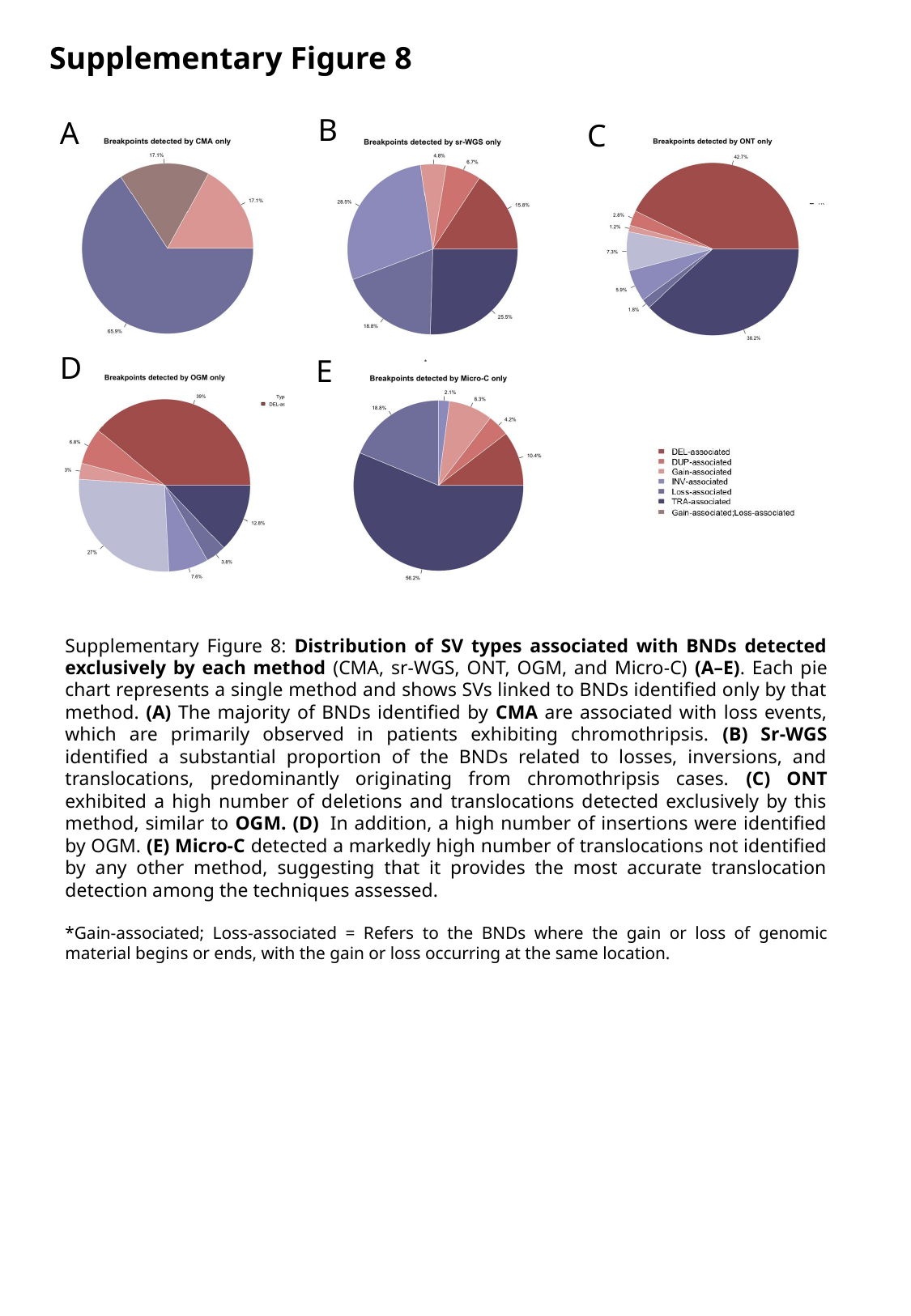

# Supplementary Figure 8
B
A
C
*
D
E
Supplementary Figure 8: Distribution of SV types associated with BNDs detected exclusively by each method (CMA, sr-WGS, ONT, OGM, and Micro-C) (A–E). Each pie chart represents a single method and shows SVs linked to BNDs identified only by that method. (A) The majority of BNDs identified by CMA are associated with loss events, which are primarily observed in patients exhibiting chromothripsis. (B) Sr-WGS identified a substantial proportion of the BNDs related to losses, inversions, and translocations, predominantly originating from chromothripsis cases. (C) ONT exhibited a high number of deletions and translocations detected exclusively by this method, similar to OGM. (D)  In addition, a high number of insertions were identified by OGM. (E) Micro-C detected a markedly high number of translocations not identified by any other method, suggesting that it provides the most accurate translocation detection among the techniques assessed.
*Gain-associated; Loss-associated = Refers to the BNDs where the gain or loss of genomic material begins or ends, with the gain or loss occurring at the same location.

## Slide 14
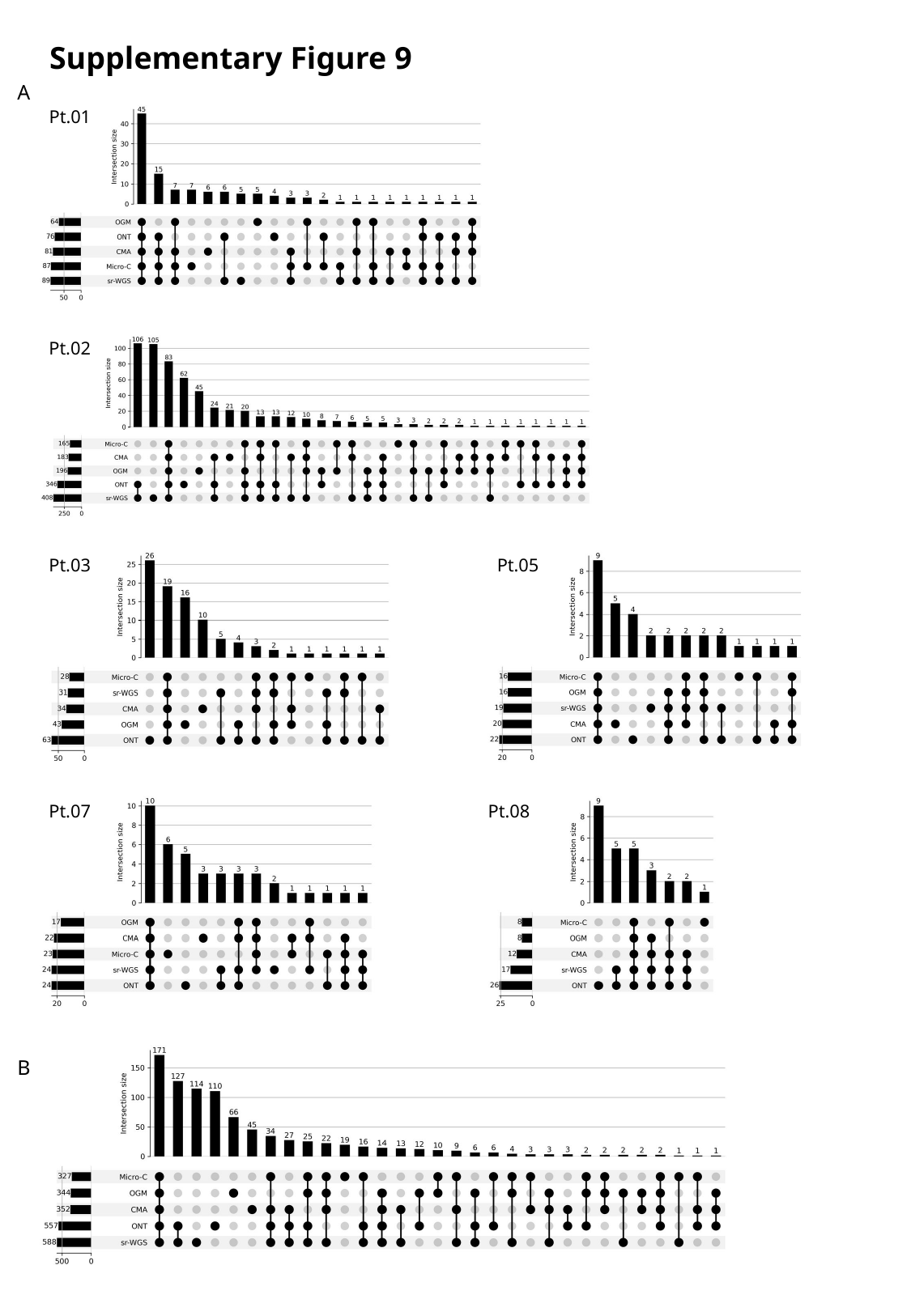

# Supplementary Figure 9
A
Pt.01
Pt.02
Pt.03
Pt.05
Pt.07
Pt.08
B

## Slide 15
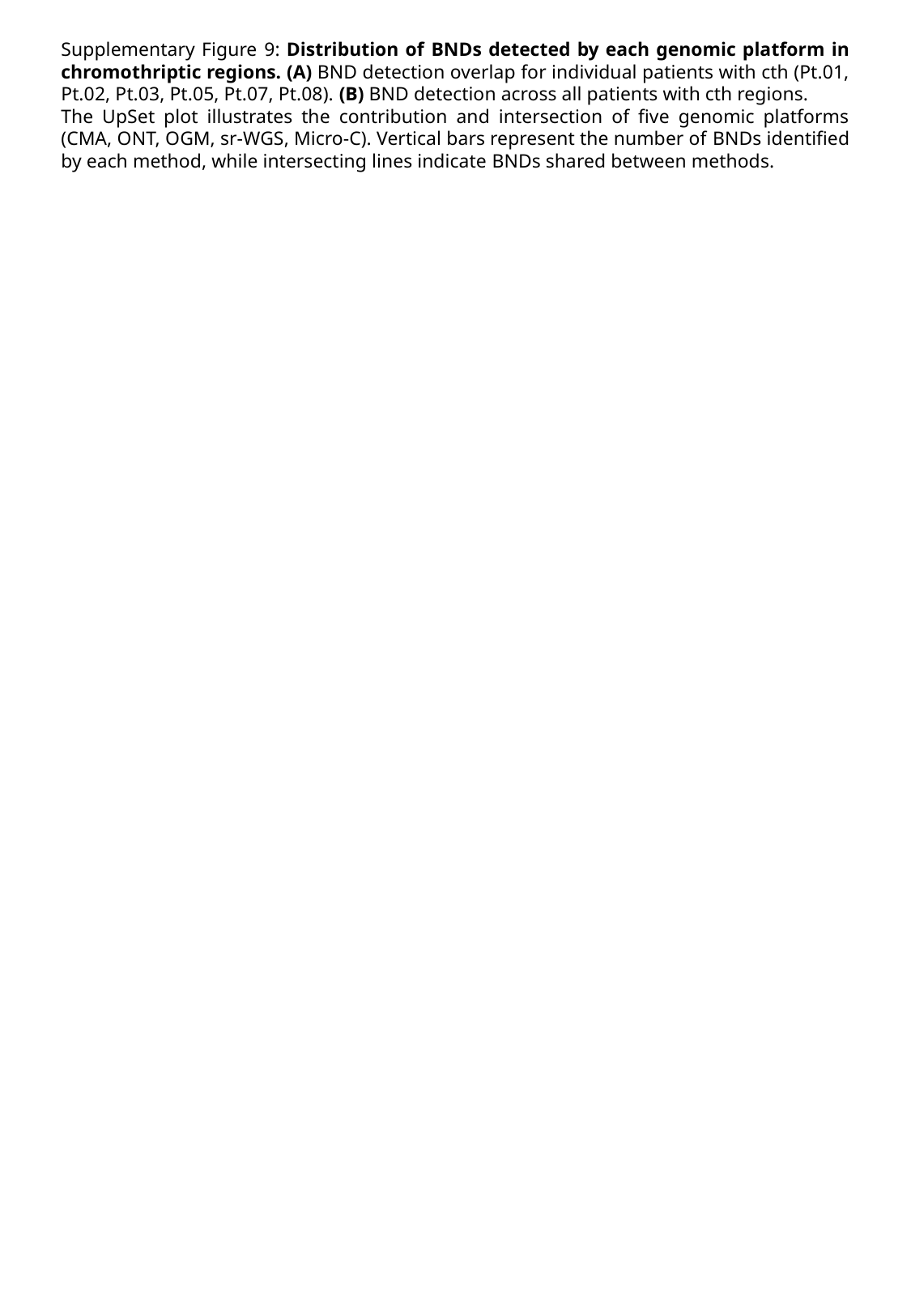

Supplementary Figure 9: Distribution of BNDs detected by each genomic platform in chromothriptic regions. (A) BND detection overlap for individual patients with cth (Pt.01, Pt.02, Pt.03, Pt.05, Pt.07, Pt.08). (B) BND detection across all patients with cth regions.
The UpSet plot illustrates the contribution and intersection of five genomic platforms (CMA, ONT, OGM, sr-WGS, Micro-C). Vertical bars represent the number of BNDs identified by each method, while intersecting lines indicate BNDs shared between methods.

## Slide 16
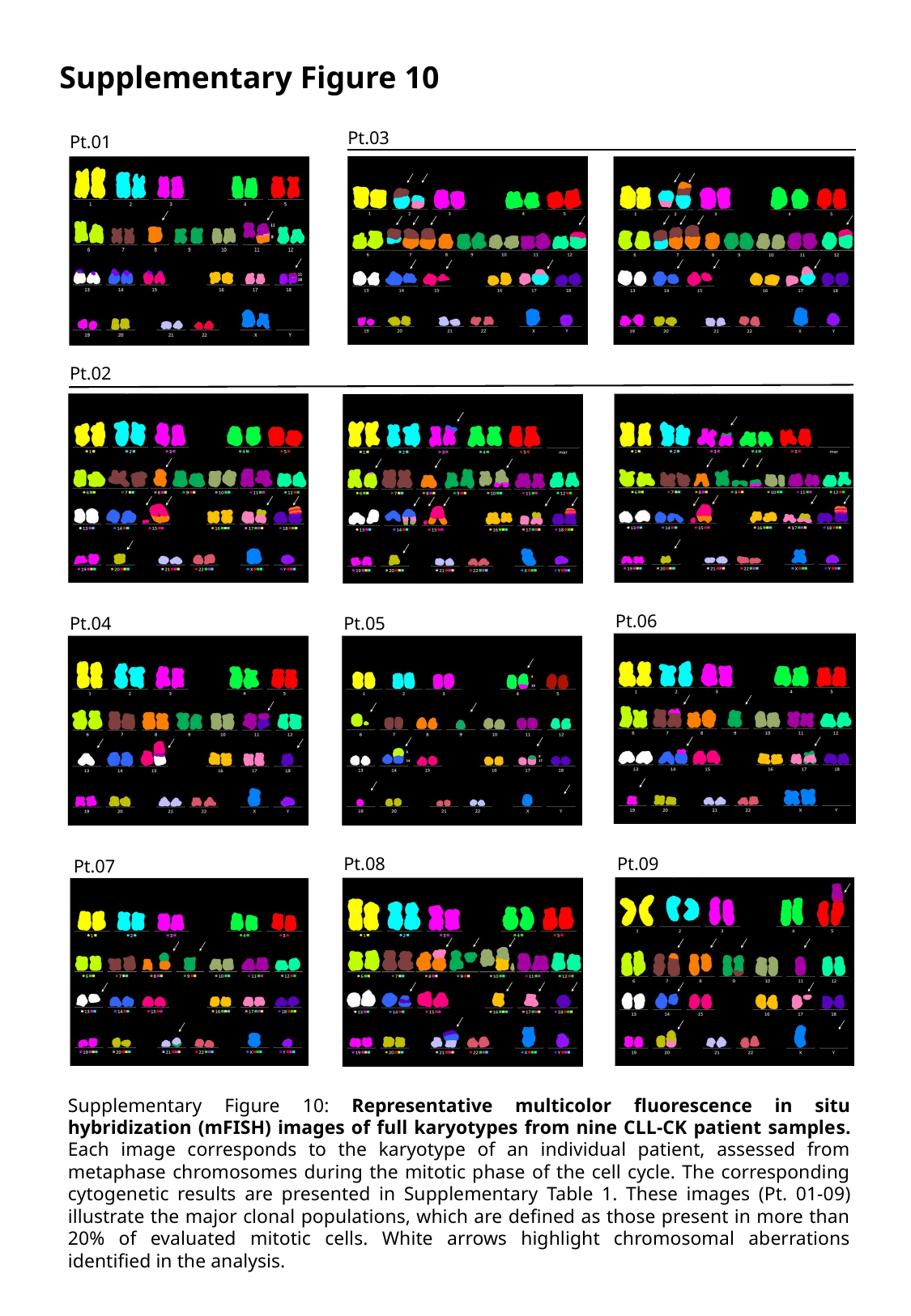

Supplementary Figure 10
Pt.03
Pt.01
Pt.02
Pt.06
Pt.04
Pt.05
Pt.08
Pt.09
Pt.07
Supplementary Figure 10: Representative multicolor fluorescence in situ hybridization (mFISH) images of full karyotypes from nine CLL-CK patient samples. Each image corresponds to the karyotype of an individual patient, assessed from metaphase chromosomes during the mitotic phase of the cell cycle. The corresponding cytogenetic results are presented in Supplementary Table 1. These images (Pt. 01-09) illustrate the major clonal populations, which are defined as those present in more than 20% of evaluated mitotic cells. White arrows highlight chromosomal aberrations identified in the analysis.

## Slide 17
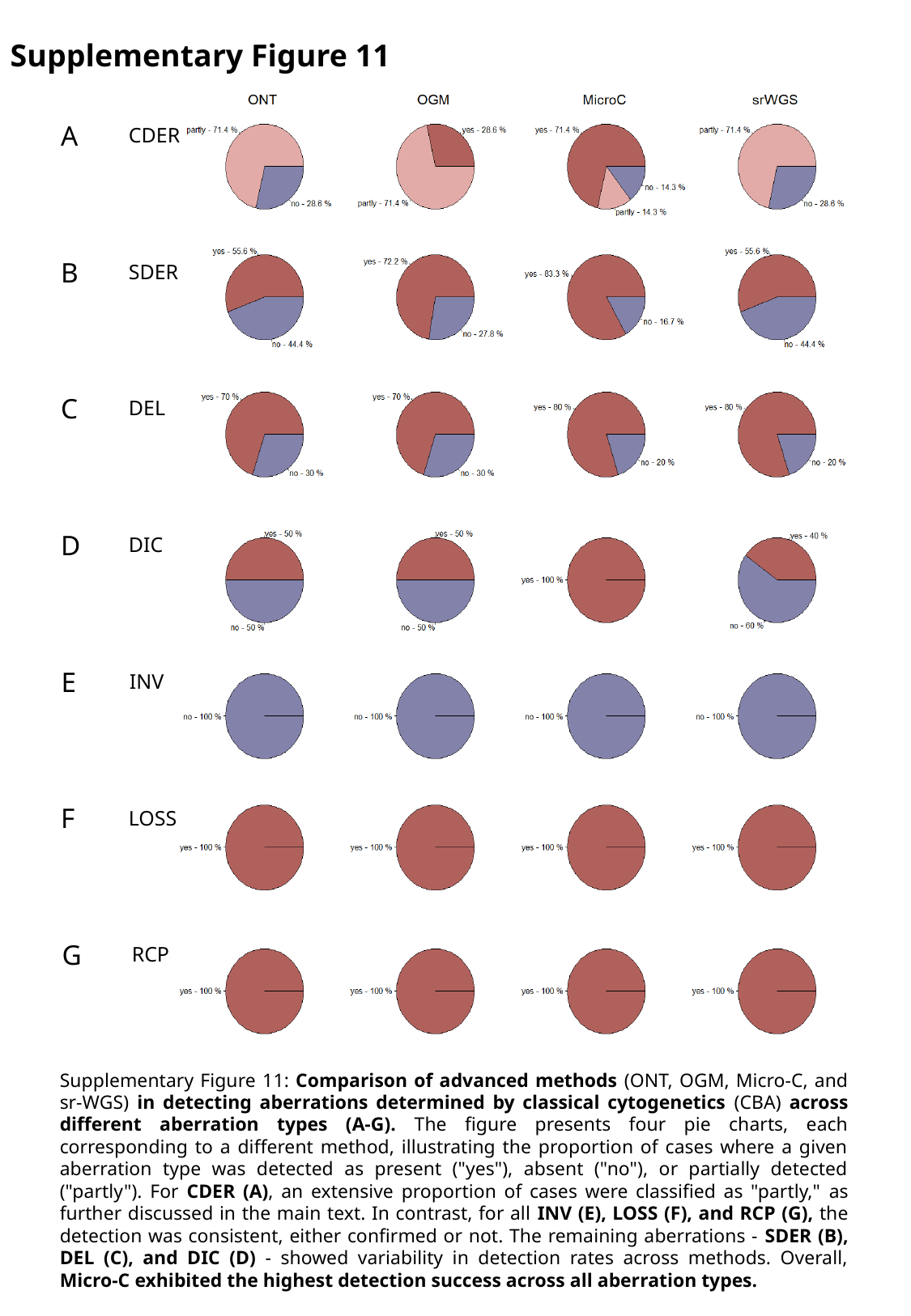

Supplementary Figure 11
A
B
C
D
E
F
G
CDER
SDER
DEL
DIC
INV
LOSS
RCP
Supplementary Figure 11: Comparison of advanced methods (ONT, OGM, Micro-C, and sr-WGS) in detecting aberrations determined by classical cytogenetics (CBA) across different aberration types (A-G). The figure presents four pie charts, each corresponding to a different method, illustrating the proportion of cases where a given aberration type was detected as present ("yes"), absent ("no"), or partially detected ("partly"). For CDER (A), an extensive proportion of cases were classified as "partly," as further discussed in the main text. In contrast, for all INV (E), LOSS (F), and RCP (G), the detection was consistent, either confirmed or not. The remaining aberrations - SDER (B), DEL (C), and DIC (D) - showed variability in detection rates across methods. Overall, Micro-C exhibited the highest detection success across all aberration types.

## Slide 18
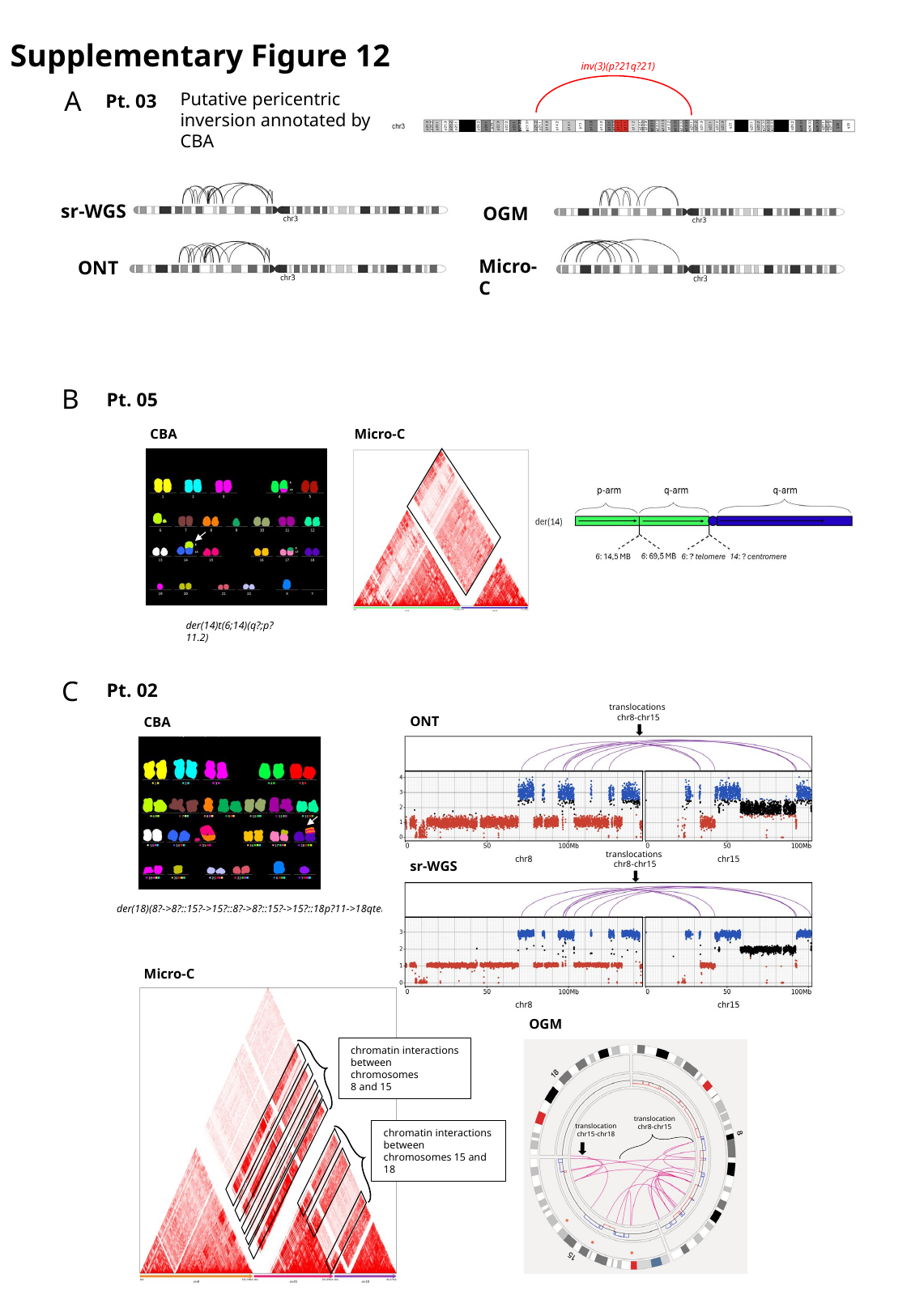

Supplementary Figure 12
inv(3)(p?21q?21)
A
Putative pericentric inversion annotated by CBA
Pt. 03
sr-WGS
OGM
Micro-C
ONT
B
Pt. 05
CBA
der(14)t(6;14)(q?;p?11.2)
Micro-C
C
Pt. 02
translocations
chr8-chr15
ONT
⬊
CBA
der(18)(8?->8?::15?->15?::8?->8?::15?->15?::18p?11->18qter)
translocations
chr8-chr15
sr-WGS
⬊
OGM
translocation chr8-chr15
translocation chr15-chr18
⬊
Micro-C
chromatin interactions between chromosomes
8 and 15
chromatin interactions between chromosomes 15 and 18

## Slide 19
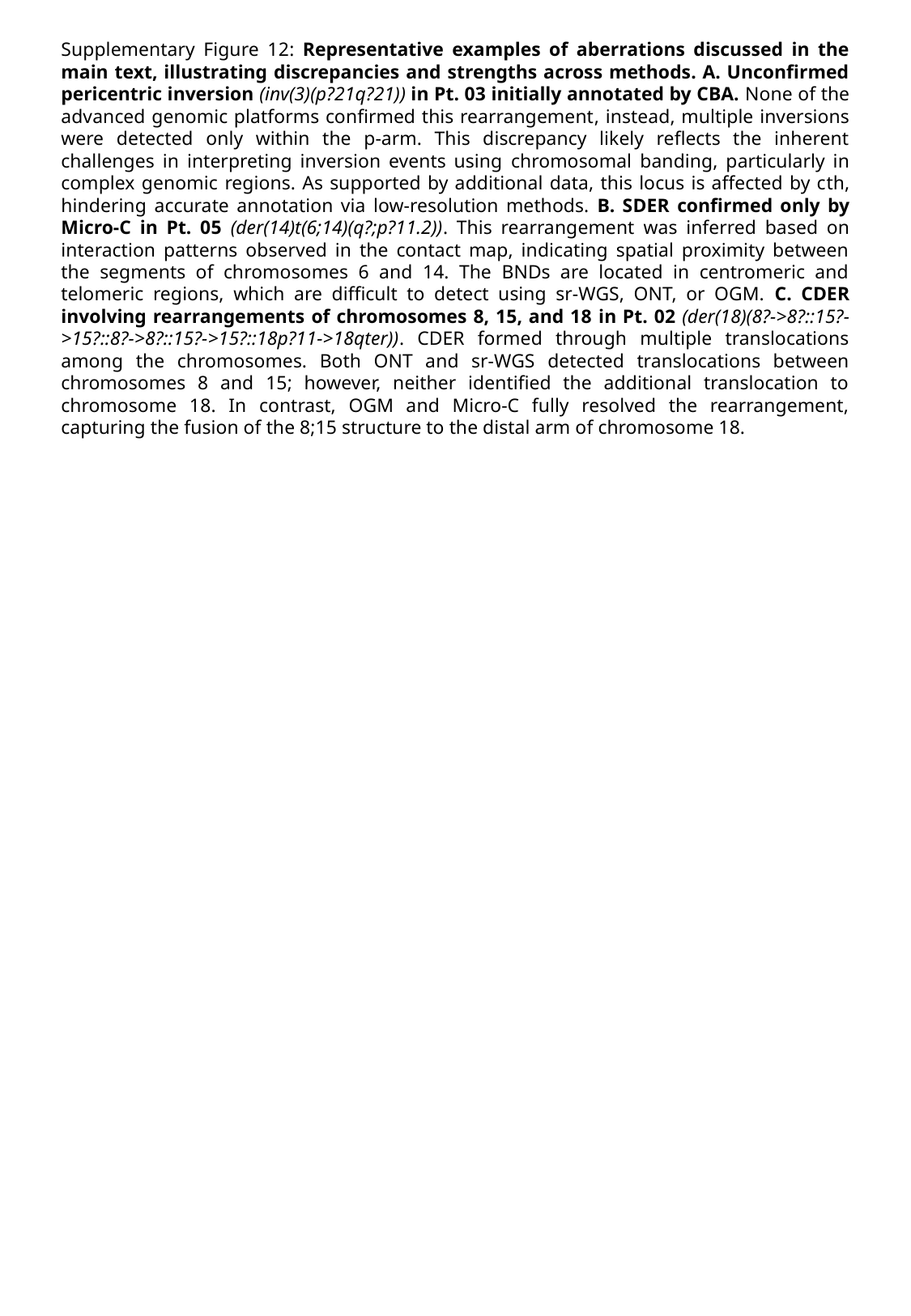

Supplementary Figure 12: Representative examples of aberrations discussed in the main text, illustrating discrepancies and strengths across methods. A. Unconfirmed pericentric inversion (inv(3)(p?21q?21)) in Pt. 03 initially annotated by CBA. None of the advanced genomic platforms confirmed this rearrangement, instead, multiple inversions were detected only within the p-arm. This discrepancy likely reflects the inherent challenges in interpreting inversion events using chromosomal banding, particularly in complex genomic regions. As supported by additional data, this locus is affected by cth, hindering accurate annotation via low-resolution methods. B. SDER confirmed only by Micro-C in Pt. 05 (der(14)t(6;14)(q?;p?11.2)). This rearrangement was inferred based on interaction patterns observed in the contact map, indicating spatial proximity between the segments of chromosomes 6 and 14. The BNDs are located in centromeric and telomeric regions, which are difficult to detect using sr-WGS, ONT, or OGM. C. CDER involving rearrangements of chromosomes 8, 15, and 18 in Pt. 02 (der(18)(8?->8?::15?->15?::8?->8?::15?->15?::18p?11->18qter)). CDER formed through multiple translocations among the chromosomes. Both ONT and sr-WGS detected translocations between chromosomes 8 and 15; however, neither identified the additional translocation to chromosome 18. In contrast, OGM and Micro-C fully resolved the rearrangement, capturing the fusion of the 8;15 structure to the distal arm of chromosome 18.
